# HIV-1 RNA Levels and Antiretroviral Drug Resistance in Blood and Non-Blood Compartments from HIV-1–Infected Men and Women enrolled in AIDS Clinical Trials Group Study A5077

**DOI:** 10.1371/journal.pone.0093537

**Published:** 2014-04-03

**Authors:** Rami Kantor, Daniel Bettendorf, Ronald J. Bosch, Marita Mann, David Katzenstein, Susan Cu-Uvin, Richard D’Aquila, Lisa Frenkel, Susan Fiscus, Robert Coombs, for the ACTG A Study Team

**Affiliations:** 1 Division of Infectious Diseases, Department of Medicine, Brown University Alpert Medical School, Providence, Rhode Island, United States of America; 2 Harvard School of Public Health, Harvard University, Boston, Massachusetts, United States of America; 3 Division of Infectious Diseases, Department of Medicine, Stanford University, Stanford, California, United States of America; 4 Division of Infectious Diseases, Department of Medicine, Northwestern University, Chicago, Illinois, United States of America; 5 Department of Pediatrics and Laboratory Medicine, University of Washington School of Medicine, Seattle, Washington, United States of America; 6 Center for Infectious Diseases, University of North Carolina, Chapel Hill, North Carolina, United States of America; 7 Department of Laboratory Medicine and Division of Infectious Diseases, Department of Medicine, University of Washington School of Medicine, Seattle, Washington, United States of America; University of Pittsburgh, United States of America

## Abstract

**Background:**

Detectable HIV-1 in body compartments can lead to transmission and antiretroviral resistance. Although sex differences in viral shedding have been demonstrated, mechanisms and magnitude are unclear. We compared RNA levels in blood, genital-secretions and saliva; and drug resistance in plasma and genital-secretions of men and women starting/changing antiretroviral therapy (ART) in the AIDS Clinical Trials Group (ACTG) 5077 study.

**Methods:**

Blood, saliva and genital-secretions (compartment fluids) were collected from HIV-infected adults (≥13 years) at 14 United-States sites, who were initiating or changing ART with plasma viral load (VL) ≥2,000 copies/mL. VL testing was performed on all compartment fluids and HIV resistance genotyping on plasma and genital-secretions. Spearman rank correlations were used to evaluate concordance and Fisher’s and McNemar’s exact tests to compare VL between sexes and among compartments.

**Results:**

Samples were available for 143 subjects; 36% treated (23 men, 29 women) and 64% ‘untreated’ (40 men, 51 women). RNA detection was significantly more frequent in plasma (100%) than genital-secretions (57%) and saliva (64%) (P<0.001). A higher proportion of men had genital shedding versus women (78% versus 41%), and RNA detection was more frequent in saliva versus genital-secretions in women when adjusted for censoring at the limit of assay detection. Inter-compartment fluid VL concordance was low in both sexes. In 22 (13 men, 9 women) paired plasma-genital-secretion genotypes from treated subjects, most had detectable resistance in both plasma (77%) and genital-secretions (68%). Resistance discordance was observed between compartments in 14% of subjects.

**Conclusions:**

HIV shedding and drug resistance detection prior to initiation/change of ART in ACTG 5077 subjects differed among tissues and between sexes, making the gold standard blood-plasma compartment assessment not fully representative of HIV at other tissue sites. Mechanisms of potential sex-dependent tissue compartmentalization should be further characterized to aid in optimizing treatment and prevention of HIV transmission.

**Trial Registration:**

ClinicalTrials.gov NCT00007488

## Introduction

Sexual transmission of the human immunodeficiency virus type-1 (HIV) is directly related to HIV RNA level in the genital tract [1], [2], [3]. Measurement of HIV RNA in the genital tract is not a convenient clinical tool, and quantification of plasma HIV RNA is used for clinical monitoring of HIV-infected subjects [4], [5]. Plasma HIV RNA correlates with viral detection in both genital fluid [6], [7], [8], [9] and saliva [10], [11]. Although anti-retroviral therapy (ART) effectively suppresses viral replication in all three compartments [9], [12], [13], [14], approximately10–40% of ART-treated subjects have detectable HIV in the genital tract fluid but not in blood plasma [6], [7], [8], [9], [15], [16], [17], [18], [19], [20], [21], [22]. Such discordances suggest a compartment-specific milieu for viral replication in blood and non-blood compartments [23], [24], which could lead to an erroneous perception of genital viral suppression and persistent potential for HIV transmission despite suppression of HIV in the blood compartment.

HIV genital shedding is sporadic and may be associated with age [6], co-infections [8], drug penetration [25], [26], hormonal levels, menstrual cycle and sex [8]. Although sex differences in genital shedding have been consistently demonstrated, the magnitude of the differences and mechanisms involved are not well understood. Women may have lower plasma HIV RNA levels compared to men with equivalent CD4 levels, whereas ART may be more successful in suppressing genital HIV RNA in men than women [26], [27], [28], [29], [30], [31], [32]. Differences in compartment-specific HIV RNA between ART-experienced men and women with suppressed plasma virus imply that men and women may not represent an immunologically or pharmacologically homogenous group, which would have implications for treatment and disease progression.

Viral replication in and shedding from the genital tract despite ART and suppression of plasma HIV RNA may increase the risk of developing drug resistance [33], [34]. Consequently, HIV tropism and resistance patterns can differ markedly among anatomic compartment sites [35], [36], [37], [38], [39], which indicates local HIV replication in the genital tract [40], [41]. We hypothesized that underlying sex differences in genital viral replication (as measured by HIV RNA level) may lead to sex differences in drug resistance evolution and thus different viral drug resistance patterns between blood and genital tract for different classes of ART drugs.

We present study enrollment data from AIDS Clinical Trials Group (ACTG) study 5077, an observational study designed to examine inter-compartmental viral differences among HIV-infected men and women to address this hypothesis. We compared HIV levels in blood plasma, genital-secretions and saliva, and HIV drug resistance in plasma and genital-secretions of men and women who were either starting or changing ART.

## Methods

### Ethics Statement

Institutional review boards at the following participating institutions approved the protocol: Johns Hopkins University, Stanford University, San Francisco General Hospital, University of Rochester, University of Southern California Medical Center, University of Washington, University of Minnesota, Washington University, The Ohio State University, Rush University Medical Center, Miriam Hospital Rhode Island, University of North Carolina, University of Hawaii at Manoa Leahi Hospital and the Columbia University Presbyterian Medical Center. A written informed consent was obtained from all participants. The ACTG A5077 protocol did allow for informed consent from parent/legal guardian on behalf of the minors/children. However, there were no subjects enrolled who were <18 years of age, so informed consent was never obtained from parents/guardians on behalf of the minors/children enrolled in our study.

### Population and Sample Collection

ACTG study 5077 was a 96-week, multi-center longitudinal study designed to evaluate viral shedding and antiretroviral drug resistance characteristics by body compartment in men and women (see Supplementary Protocol S1). The study was conducted between March 2001 and October 2002. Eligible HIV-infected subjects age ≥13 years from 14 United-States ACTG sites were offered enrollment if they: (1) were initiating or changing highly active ART (HAART); (2) had plasma HIV RNA ≥2,000 copies/mL within 60 days prior to study enrollment; and (3) were willing to contribute samples of blood, saliva and genital-secretions. HAART was defined as a minimum of three antiretroviral drugs that included any of the following six combinations: (i) two nucleoside reverse transcriptase inhibitors (NRTIs) and one non-NRTI (NNRTI); (ii) two NRTIs and abacavir; (iii) two NRTIs and a protease inhibitor (PI); (iv) two PIs and an NNRTI; (v) two PIs and a NRTI; or (vi) a NRTI, a NNRTI and a PI. Subjects were excluded if they: (1) were pregnant; (2) had active opportunistic infections (OIs), intercurrent illnesses, or other infections within 14 days prior to study enrollment; (3) had used any immunomodulatory agents within 14 days prior to study enrollment; or (4) had any active immunization within 14 days prior to study enrollment.

Upon obtaining informed consent, clinic records were reviewed for demographic information, history of HIV-related diagnoses and AIDS-defining events, and ART history. Samples obtained included blood, saliva, and genital-secretions. Specimens for plasma HIV RNA levels and blood CD4+ cell counts were obtained at entry and every 8 weeks thereafter for 96 weeks, as well as at “virological failure,” which was defined as a confirmed detection of ≥200 RNA copies/mL ≥16 weeks after starting or changing ART. Compartmental fluid samples were obtained at baseline, weeks 16, 48 and 96, and at “virological failure”.

Saliva samples were obtained by expectoration into a 50-mL conical centrifuge tube that was then frozen at −20°C within 2 hours of collection and stored on site at −80°C. Genital-secretions for men consisted of seminal fluid collected via masturbation into a sterile urine container, following 48-hours of abstinence. For women, a pelvic examination was performed with collection of three endocervical wicks (Sno-strips, Chauvin Pharmaceuticals Limited, Essex, UK) [42]. Following a one to three minute wicking period, the three wicks were removed, cut at the wick neck, placed into 500 μL of NASBA Buffer solution (bioMerieux, Durham, NC) and stored at −70°C. Women refrained from any kind of sexual activity, douching or insertion of intravaginal products for at least 48 hours prior to the collection of cervical specimens. Cervical vaginal lavage (CVL) samples were collected by bathing the cervix and ectocervix three-times with the same 10-mL of phosphate-buffered-saline solution, which was then aspirated and placed into a sterile 50-mL conical centrifuge tube. If signs or symptoms of a genitourinary tract infection were noted, the subject was evaluated, diagnosed and treated appropriately for at least 14 days and the infection considered resolved prior to genital-secretion sampling. Subjects were not screened for asymptomatic genital tract infections.

Genital specimens were processed within four hours of collection and stored on site at −70°C prior to shipment on dry ice to the ACTG central specimen repository (Biomedical Research Institute (BRI), Rockville, MD USA) for long-term storage at −80°C. Frozen specimen aliquots were shipped to Johns Hopkins University for HIV-1 RNA testing of blood plasma; to the University of North Carolina for HIV RNA testing of semen and saliva; and to the University of Washington ACTG Virology Specialty Laboratory for HIV RNA quantification in cervical and cervicovaginal fluids.

### Laboratory Testing

HIV RNA was measured in blood plasma using RT-PCR (Monitor version 1.5 (Roche Molecular) at a central laboratory. HIV RNA was extracted into NucliSens extraction buffer from saliva, seminal plasma or endocervical wicks and quantified using published NucliSens (bioMerieux; saliva, seminal plasma) or real-time RT-PCR assay methods [43], [44], [45], [46]. The lower limits of viral RNA detection were 50 copies/mL in blood plasma, 400 copies/mL in saliva, 400 copies/mL in semen plasma, 1,500 copies/mL in cervical Sno-strip fluid, and 75 copies/mL in CVL fluid. The difference in the lower limit of detection between seminal plasma and endocervical wicks was due to the requirement for sample dilution during specimen processing. The intra-assay standard deviation was <0.3 log_10_ RNA copies/mL of seminal plasma from men, based on blinded testing [44]. A similar intra-assay standard deviation was obtained for HIV-1 RNA measurements from endocervical wicks and CVL, but these data were generated from a previous assay validation for a longitudinal genital tract HIV-1 shedding study of women [43], [47].

HIV RNA from blood plasma and genital-secretions was sequenced through the *gag/pol* genes protease and reverse transcriptase regions at four ACTG virology laboratories that participated in the NIAID-DAIDS-sponsored Virology Quality Assurance (VQA) Program for HIV genotyping, including Vanderbilt University (VU), University of North Carolina (UNC), Stanford University (SU) and University of Washington (UW). Paired blood and genital-secretion samples were run at the same labs. Three different methods were used for genotyping including TRUGENE HIV Genotyping Kit for RT-PCR amplification and OpenGene System for sequencing (Siemens Health Care) at VU and UNC; Invitrogen RT-PCR and sequencing at Stanford University, and an in-house independently validated assay for amplification and ABI 377 sequencing at UW.

The raw nucleotide sequence data were sent for analyses to the Frontier Science & Technology Research Foundation (FSTRF) data management center and to the Statistical Data Analysis Center (SDAC) of the ACTG. Quality control of sequence data was performed via phylogenetic analysis using PHYLIP 3.65. The sequences have been deposited in Genbank under accession numbers KJ468189–KJ468232.

### Statistical Analysis

Patients were allocated into ‘untreated’ and treated groups for analysis. ‘Untreated’ for this study was defined as having had no prior ART exposure ever or for at least 90 days or more (off treatment) prior to study enrollment, according to patient self-report. HIV RNA level was assessed in both the ‘untreated’ and treated patient groups, while drug resistance was evaluated only in the treated group, which was expected to have resistant and/or wild-type viruses.

Spearman rank correlations were used to evaluate concordance between compartment sites. Fisher’s exact test was used to compare HIV RNA level between men and women in saliva and genital tract, and Wilcoxon rank-sum tests were used to compare HIV RNA levels in the blood between men and women. HIV RNA detection was compared between compartments using McNemar’s test. Patients were considered to be “shedders” if HIV RNA was detectable in the compartment fluid. Left-censored HIV RNA levels (i.e., measurements below assay limits) were analyzed as the lowest rank. For women with both Sno-strip and CVL samples available, Sno-strip results were analyzed because they were more precise [47].

Drug resistance mutations were assessed at protease and reverse transcriptase resistance-related positions in plasma and genital tissues in men and women according to the Stanford HIV Sequence Database tools (http://hivdb.stanford.edu; accessed 4/16/2013). Sequence analyses were restricted to treated subjects (i.e. those taking ART at or within 90 days of study enrollment) since resistance mutations can fade in the absence of antiretroviral drug pressure [48]. Resistance was measured as overall (i.e. any resistance mutation), drug class, and specific mutations. Plasma-genital tract concordance of resistance mutations was assessed and compared between sexes. Resistance discordance was defined as at least one drug resistance amino acid mutation that occurred in one compartment and not the other within a plasma and genital-secretion sequence pair. Amino acid mixtures including a resistance mutation were classified as mutant.

## Results

### Patient Characteristics

**Figure 1:**
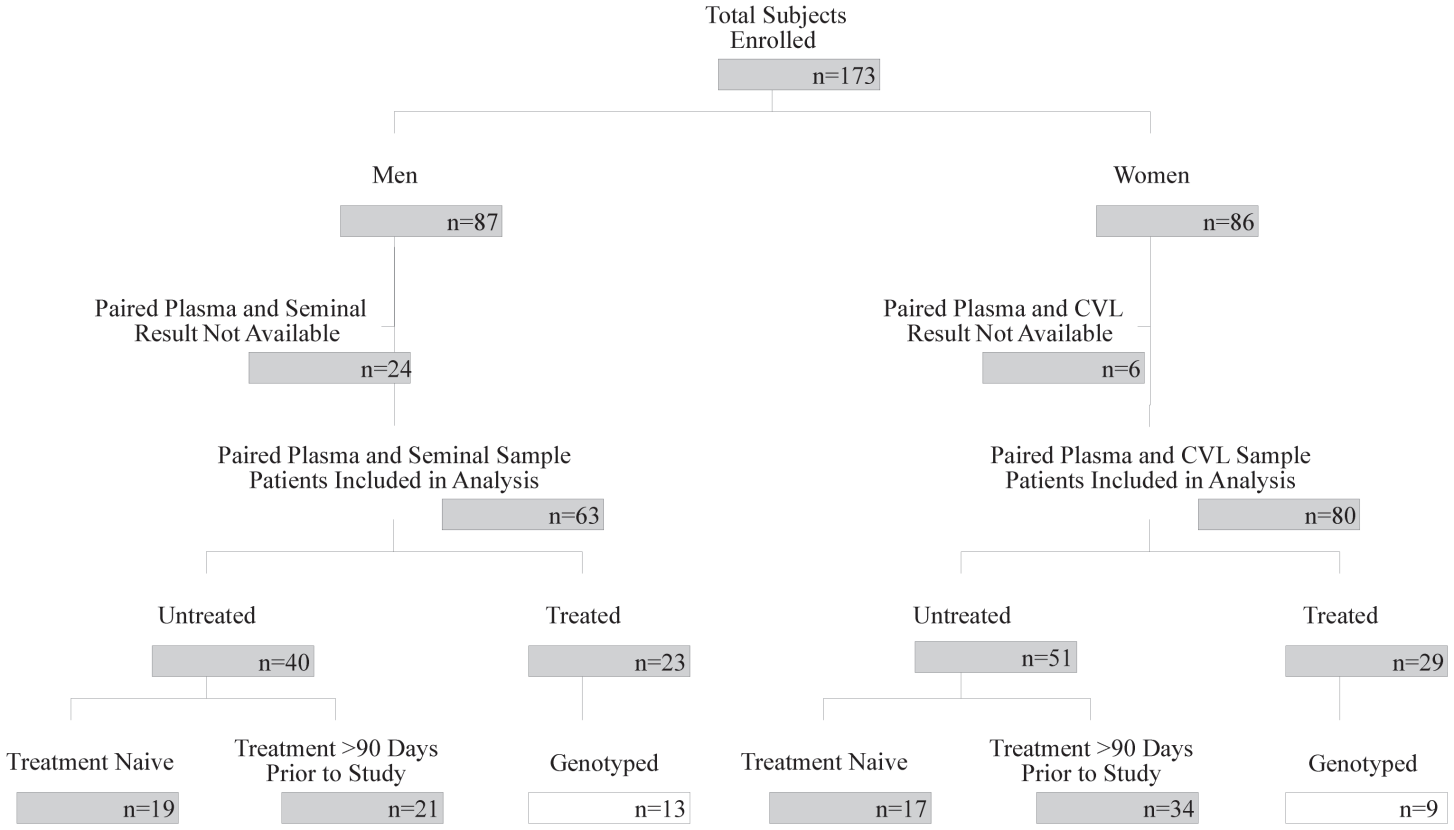
Flowchart of Enrolled Subjects and Available Samples by Sex. White boxes represent the subset of subjects with genotypes available from both blood and genital secretions. The ‘untreated’ group refers to subjects who were either antiretroviral drug naïve or who had been off ART for ≥90 days.

Between March 2001 and October 2002, 173 patients (87 men and 86 women) were enrolled (Figure 1). Of these, 143 (63 men and 80 women) had available paired blood plasma and genital compartment VL measurements, and were included in the analysis. Fourteen men and five women chose not to provide genital samples. Ten seminal, 11 Sno-strips and one CVL samples were not successfully evaluated because of insufficient specimen volume or unsuccessful PCR amplification. Among these 143 patients, 52 (36%) were treated (23 men, 29 women), and 91 (64%) were ‘untreated’ (40 men, 51 women). Of the ‘untreated’, 19 men and 17 women were treatment naïve and the remainders were not exposed to ART within 90 days prior to study enrollment. Of women, 83% of treated and 90% of ‘untreated’ had Sno-strips available in addition to CVL. Paired saliva samples were available for 88% of patients, 89% of women and 87% of men.

Patient characteristics at study enrollment are shown in Table 1 by sex and treatment history. Median age was 43 years in treated patients and 39 in ‘untreated’. Treated patients had taken a median of seven different antiretroviral drugs prior to enrollment, whereas ‘untreated’ patients were exposed to a median of three (per definition, >90 days prior to study enrollment). Among treated subjects, the most common regimens at study enrollment included NRTIs and PIs (29%) or NRTIs, NNRTIs and PIs (29%). Treated subjects had a median of 6.2 years since first ART use, and ‘untreated’ subjects had a median of 3.2 years. Median CD4 count at enrollment was 197 cells/μL in treated subjects and 216 cells/μL in ‘untreated’. There were no significant differences between men and women in HIV RNA level or CD4 count, overall or within the treated and ‘untreated’ groups (p>0.1).

**Table 1:** Characteristics of Enrolled Subjects at Study Enrollment by Sex and History of ART.

	Male		Female		All	
	n = 63		n = 80		n = 143	
	Treated	Untreated >90 days	Treated	Untreated >90 days	Treated	Untreated >90 days
	n = 23	n = 40	n = 29	n = 51	n = 52	n = 91
Age, median (IQR) years	44 (41,47)	38 (35,44)	42 (37,46)	39 (35,45)	43 (40,47)	39 (35,44)
Number of drugs ever taken, median (IQR)						
Total	8 (5,9)	2 (0,5)	7 (5,9)	4 (0,7)	7 (5,9)	3 (0,6)
NRTIs	4 (3,5)	1 (0,3)	4 (3,5)	3 (0,4)	4 (3,5)	2 (0,4)
NNRTIs	1 (0,1)	0 (0,1)	1 (1,1)	0 (0,1)	1 (1,1)	0 (0,1)
PIs	2 (1,4)	0 (0,2)	2 (1,4)	1 (0,2)	2 (1,4)	1 (0,2)
Regimen before enrollment (n)						
NRTI, NNRTI, & PI	4	–	4	–	8	–
NRTI & PI(No NNRTI)	3	–	5	–	8	–
NRTI & NNRTI(No PI)	1	–	3	–	4	–
Other	5	–	3	–	8	–
None	10	–	14	–	24	–
Years since first ARV,	7.3	0.8	5.8	4.4	6.2	3.2
use median (IQR) years	(4.6, 11.5)	(0, 5.4)	(3.8, 7.8)	(0, 6.6)	(4.1, 9.5)	(0, 6.4)
CD4 cell count/μL,	185	241	210	183	197	216
median (IQR)	(108, 314)	(121, 334)	(76, 401)	(87, 284)	(96, 361)	(93, 294)
Plasma HIV RNA,	4.6	4.8	4.4	4.8	4.6	4.8
median log_10_ c/mL (IQR)	(4.4, 5.1)	(4.5, 5.6)	(4.1, 5.1)	(4.3, 5.4)	(4.1, 5.1)	(4.4, 5.5)
Number of Subjects with Samples Collected						
Plasma	23	40	29	51	52	91
Semen	23	40	–	–	23	40
Sno-strip	–	–	24	46	24	46
CVL	–	–	29	51	29	51
Saliva	19	36	25	46	44	82

Footnote: n, number; IQR, interquartile range; NRTI, nucleoside reverse transcriptase inhibitor; NNRTI, non-nucleoside reverse transcriptase inhibitor; PI, protease inhibitor; ARV, antiretroviral; CVL, cervical vaginal lavage.

### Viral RNA Level in Blood, Genital Compartments and Saliva

**Figure 2:**
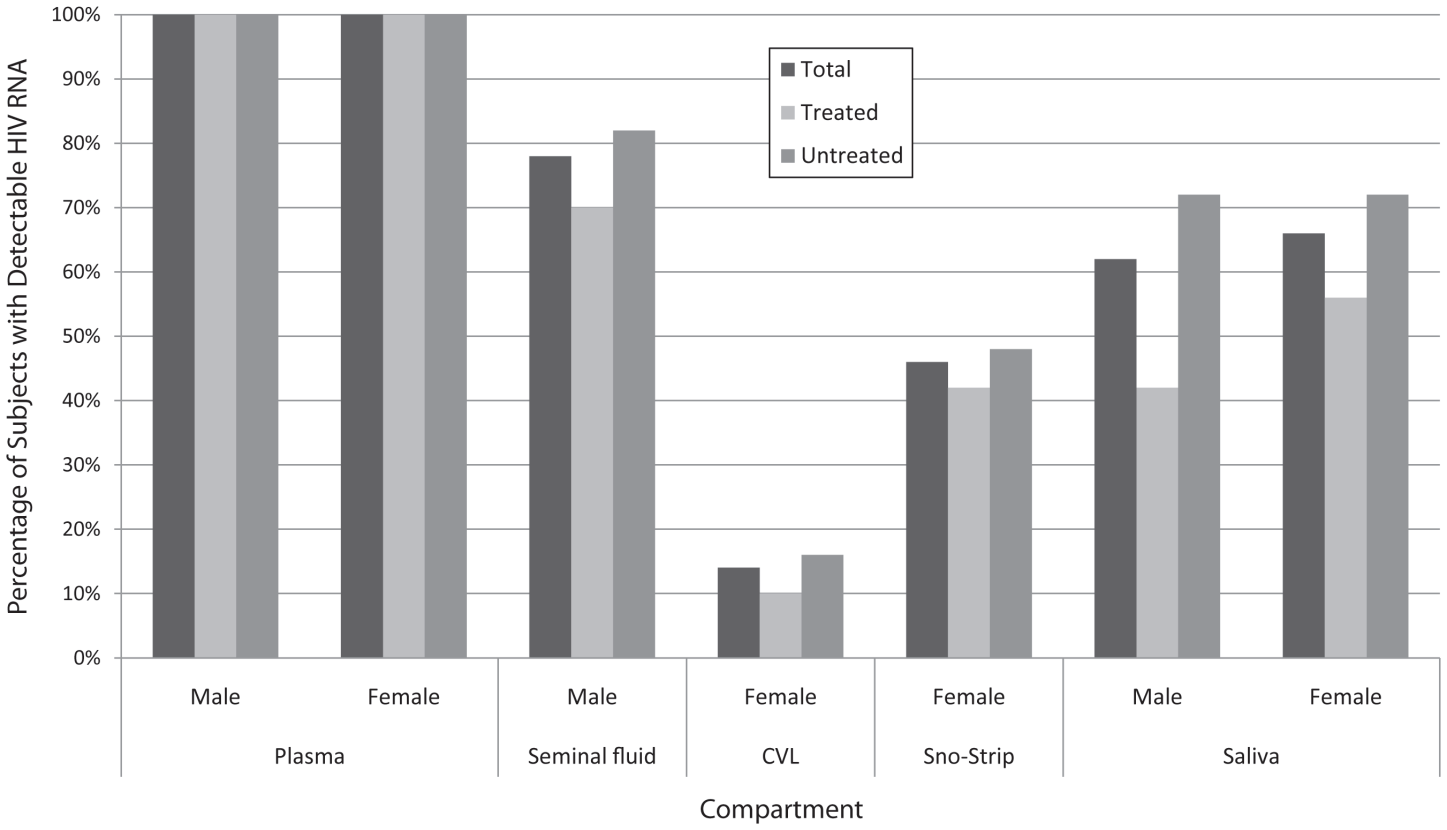
HIV RNA level in Anatomic Compartments of Enrolled Men and Women. Vertical bars represent percent of subjects with detectable viral load at study enrollment by anatomic compartment fluid and treatment status. The ‘untreated’ group refers to subjects who were either antiretroviral drug naïve or who had been off ART for ≥90 days.

Figure 2 demonstrates HIV RNA detection and concordance by sex, anatomic site, and treatment status. Overall viral RNA detection in the study cohort was 100% in blood plasma (inclusion criterion), 57% in genital-secretions (82/143) and 64% in saliva (81/126). Detection of HIV RNA in plasma was significantly more frequent than in genital-secretions and saliva (p<0.001). Sex-specific viral detection for the genital fluid was 78% (49/63) in men and 41% in women [14% (11/80) in CVL and 46% (32/70) in Sno-strip specimens] and for saliva, 62% (34/55) in men and 66% (47/71) in women. Men had significantly more frequent genital HIV RNA detection compared to women, a trend that held in both treated (70% vs. 38%, p = 0.03) and ‘untreated’ (83% vs. 43%, p<0.001) subjects. Women had HIV detected more frequently in saliva compared to genital-secretions (66% vs. 41%, p = 0.01).

**Figure 3:**
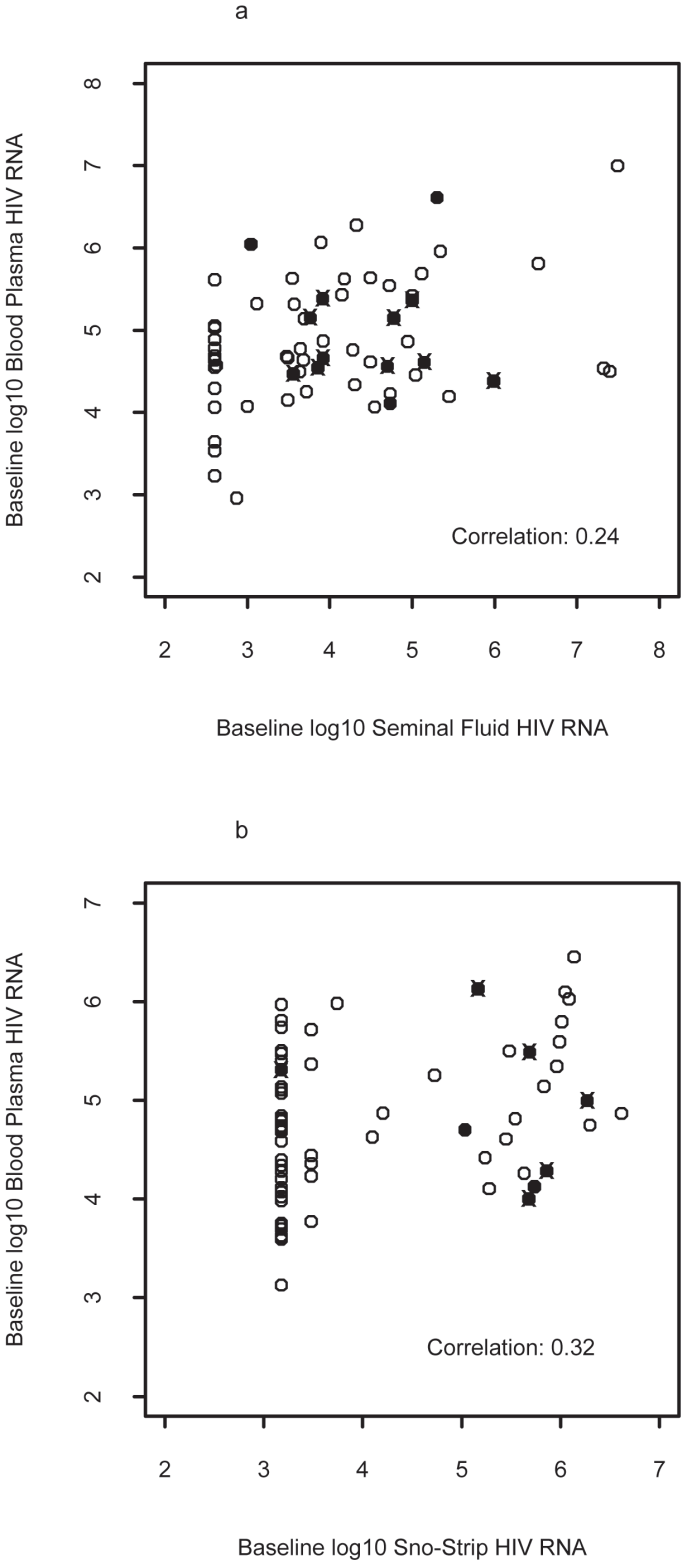
Association of Blood-Plasma and Genital Tract Fluid HIV RNA by Sex. Scatterplots demonstrating the Spearman correlations between blood and seminal plasma (A) and between blood and SnoStrip (B) viral load. Filled symbols represent those with paired blood and genital secretion genotypes; individuals with detectable drug resistance mutations in either blood or genital secretions are marked with an ‘x’.

Median plasma HIV RNA level in treated, ‘untreated’ and overall was 4.6, 4.8 and 4.7 log_10_ RNA copies/mL in men and 4.4, 4.8 and 4.7 log_10_ RNA copies/mL in women, respectively (p = 0.50 comparing all). Paired HIV RNA values were lower in all non-plasma compartments compared to plasma (Figure 2). Concordance of HIV RNA levels between blood plasma and genital-secretions was low and not significant in men (r = 0.26, 0.21 and 0.24 for treated, ‘untreated’ and overall; Figure 3a), and low but reaching statistical significance (p<0.05) for ‘untreated’ and overall in women (r = 0.20, 0.37 and 0.32 for treated, ‘untreated’ and overall; Figure 3b). For both men and women, detection of HIV RNA in genital fluids was infrequent when the plasma viral RNA level was <4.0 RNA log_10_ copies/mL.

HIV RNA level concordance between plasma and saliva was low for both men (r = 0.00, 0.38 and 0.25 for treated, ‘untreated’ and overall) and women (r = 0.38, 0.14 and 0.30 for treated, ‘untreated’ and overall), although significant for ‘untreated’ men and women overall (p<0.05).

### Drug Resistance

Paired plasma and genital-secretion genotypes were available for 22 treated subjects, 13 men and 9 women (2/9 CVL and 7/9 Sno Strips) (Figure 1). All sequences were HIV-1 subtype B, and sequences from different anatomic compartments within the same patient clustered together with high (>90%) bootstrap support.

**Figure 4:**
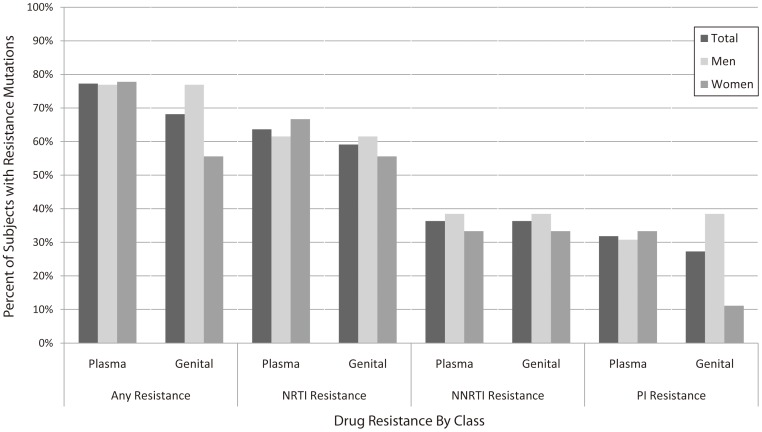
Antiretroviral Drug Resistance in Plasma and Genital Tract Fluid by Drug Class in Enrolled Men and Women. Vertical bars represent percent of subjects with resistance mutations, overall and by drug class (NRTI: nucleoside reverse transcriptase inhibitor, NNRTI: non-nucleoside reverse transcriptase inhibitor, PI: protease inhibitor).

Most subjects had detectable drug resistance mutations in plasma (77%) and genital-secretions (68%); see Figure 4. More subjects had NRTI-associated mutations (64% in plasma, 59% in genital-secretions) than NNRTI (36% in plasma, 36% in genital-secretions) or PI (32% in plasma, 27% in genital-secretions)-associated mutations.

**Figure 5:**
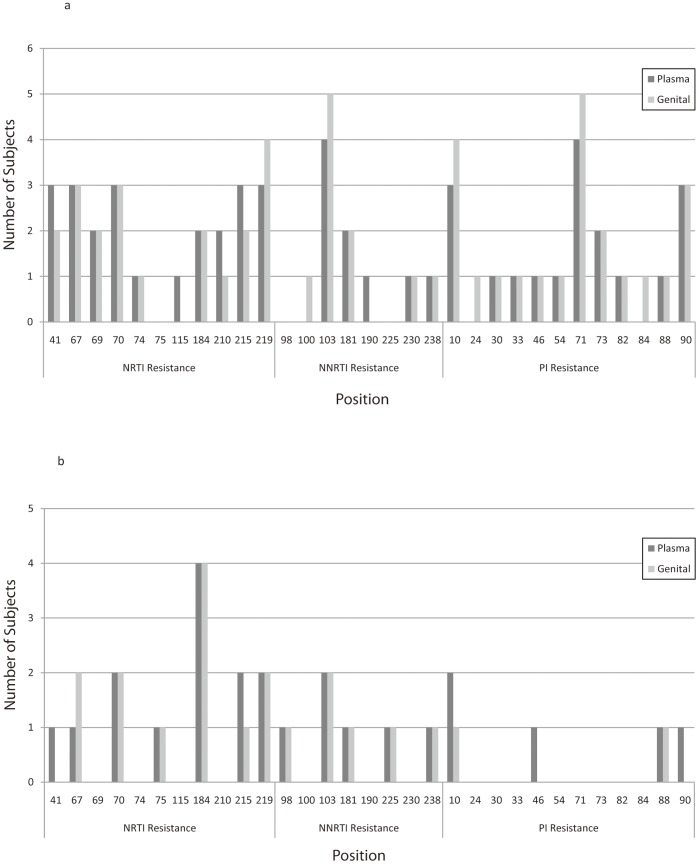
HIV Drug Resistance Mutations in Paired Plasma and Genital Tract Fluid of Enrolled Men (A) and Women (B). Vertical bars represent number of subjects with specific mutations in plasma (dark bars) and genital tract (gray bars). The X-axis depicts nucleoside reverse transcriptase inhibitor (NRTI; positions 41–219), non-nucleoside reverse transcriptase inhibitor (NNRTI; positions 98–238) and protease inhibitor (PI; positions 10–90) associated positions.

The inter-compartment concordance of drug resistant mutations varied by drug class for both men and women. In men (Figure 5a), mutations occurred in ≥3 subjects at NRTI-associated positions 41, 67, 70, 215 and 219 in plasma, and 67, 70 and 219 in semen; at NNRTI-associated position 103 in both plasma and semen; and at PI-associated positions 10, 71 and 90 in both plasma and semen. In women (Figure 5b), mutations occurred in ≥2 subjects at NRTI-associated positions 70, 184, 215 and 219 in plasma, and 67, 70, 184 and 219 in genital-secretions; at NNRTI-associated position 103 in both plasma and genital-secretions; and at PI-associated position 10 in plasma.

Based on paired plasma and genital genotypes, 7/13 (54%) men and 5/9 (56%) women demonstrated complete concordance in positions showing drug resistance. In the seven subjects (4 men, 3 women) with discordant NRTI-associated mutations in plasma versus genital-secretions, five had NRTI mutations in plasma not seen in genital-secretions; two had NRTI mutations in genital-secretions that were not detected in plasma. In the three subjects (all men) with discordant NNRTI-associated mutations, one had NNRTI mutations in plasma not seen in genital-secretions; two had NNRTI mutations in genital-secretions that were not detected in plasma. In the three subjects (1 man, 2 women) with discordant PI-associated mutations, two had PI mutations in plasma not seen in genital-secretions; one had PI mutations in genital-secretions that were not detected in plasma.

In plasma, 36% (8/22) of subjects had resistance to one ART class (38% of men, 33% of women); 27% (6/22) to two classes (23% of men, 33% of women); and 14% (3/22) to three classes (15% of men, 11% of women). In genital-secretions, 27% (6/22) of subjects had resistance to one ART class (31% of men, 22% of women); 27% (6/22) to two classes (31% of men, 22% of women); and 14% (3/22) to three classes (15% of men, 11% of women). The classes of ART with detectable resistance were the same in plasma and genital-secretions for the majority of subjects (86%, 19/22); class resistance differed for one man (NNRTI resistance in plasma vs. NNRTI and PI resistance in genital-secretions) and two women (PI resistance in plasma vs. no resistance detected in genital-secretions; NRTI and PI resistance in plasma vs. no resistance detected in genital-secretions).

## Discussion

We present inter-compartment HIV RNA detection and drug resistance comparisons among HIV-infected men and women with detectable plasma HIV RNA levels, who were initiating or changing ART in ACTG study 5077. This study, which was done in the US, enrolled equal numbers of women and men who contributed significant number of blood, genital tract and salivary samples. Many studies have enrolled only men or women and few had the ability to make significant comparisons in genital tract HIV RNA level by sex. In this study population, genital-secretion (57%) and saliva (64%) viral detection was high, and men had significantly greater detection of genital tract HIV RNA than did women. In this study population, both men and women continued to shed HIV RNA in the genital tract, thus increasing their potential risk of sexually transmitting HIV. Since a plasma HIV RNA of ≥2,000 copies/mL was an inclusion criterion, we were not able to assess genital tract HIV shedding among those with undetectable plasma viral levels. However, 21% of men and 24% of women had genital tract HIV RNA greater than plasma HIV RNA, including two men with a 2 log_10_ higher genital tract HIV RNA than plasma [49], [50]. When adjusted for the left-censoring associated with different lower limits of detection for each of the three samples assayed, concordance of HIV RNA levels between plasma and non-plasma compartments was low in both sexes, though higher in women.

HIV RNA results from this study support prior reports of frequent discordance between blood and genital compartments, although previous studies found more frequent detection of HIV in genital fluids compared to plasma, which may be in part due to the blood plasma HIV RNA ≥2000 copies/mL screening criterion in our study [10], [11], [33], [35], [51]. As inclusion criteria in this study required detectable HIV RNA in plasma, previous results could not be directly compared. Our study also supports prior findings of differences in genital detection of HIV and blood/non-blood compartmental concordance across sexes [26], [27], [28], [29], [30], [31]. However, results presented here indicate higher genital HIV RNA levels in men than women, which does not concur with one study in which men were more likely than women to suppress genital VL [52]. Baeten et al. and others have shown a direct relationship between the risks of HIV sexual transmission and genital tract HIV RNA level [2]. Regardless of the differences, HIV RNA level discordances imply that plasma HIV RNA is not fully representative of genital and other compartmental viral shedding.

HIV shedding in saliva was at higher frequency than genital HIV shedding for women but was similar for men. Though not considered a common mode of transmission [53], [54], and despite reports of antiviral activity of salivary components [55], relatively higher HIV RNA in saliva of women may represent unique, sex-dependent transmission risk associated with saliva; the transmission risk associated with oral-sex is low and still controversial. Such findings may have implications for HIV transmission in special circumstances, such as high viral replication and mandates further investigation [46], [56], [57], [58].

Most treated study subjects had drug resistance to ART detected in both plasma and genital compartments, most likely because this patient group had been somewhat adherent as opposed to completely non-adherent to ART. Our study is consistent with prior findings of compartmental differences in drug resistance [40], [41] in subjects on failing regimens. Concordance of resistance mutations between plasma and genital-secretions in both men and women varies widely across studies, ranging up to 84% [59]. Although most drug resistance mutations were identified in genital-secretions and plasma, some inter-compartmental discordance was observed. This might reflect variable drug penetration into different tissues, which may differ between men and women; e.g., the effect of the menstrual cycle on genital viral shedding [43], [60]. Importantly, discordance implies that plasma cannot be considered fully representative of drug resistance patterns in other anatomic sites. Resistance mutations in genital fluids and not in plasma were seen in our study in 14% of men and women, including mutations like RT D67N and K103N; and protease I84V, which could go unnoticed and subsequently be transmitted. Since most of this discordance occurred among subjects not on ART, it is possible that off-drug decay rates may have differed between compartments and contributed to this discordance. Based on the satisfactory participation of all four resistance-testing laboratories in an external quality assurance program for HIV genotypic drug resistance, inter-laboratory variation is less likely to explain this discordance. Nevertheless, better assays and further research are needed to examine drug resistance evolution between blood and non-plasma compartments [61].

This study has seven key limitations. First, treatment history was provided by self-report and may not be accurate or complete. This disallows the reliable determination of regimen-based resistance profiles. Second, small sample size did not allow for robust effect estimates, particularly in subjects with genotypes available. Third, different methods in different laboratories with varying detection capabilities in the respective compartment fluids because of dilution effects were used to measure HIV RNA, which increases difficulty in interpretation of comparisons, especially comparisons between male and female genital-secretions, even when all the assays were independently validated and externally quality controlled. Fourth, the cohort was not homogeneous in terms of treatment history, which can affect viral RNA levels and resistance concordance among the anatomic sites. Fifth, co-infections such as cytomegalovirus (CMV) were not evaluated. CMV in particular has been associated with HIV shedding in the male genital tract [62] and local CMV reactivation could have been a contributing factor to disproportionately high genital viral RNA levels in a subset of study participants with low CD4 cell counts and greater immune suppression. Sixth, the input viral template was not quantified or standardized for genotyping, which may have limited the detection of mutant viruses to a variable degree within and between anatomic sites. Finally, detectable plasma HIV RNA ≥2000 copies/mL was part of the inclusion criteria, and so this analysis does not include subjects with lower or undetectable plasma HIV RNA but detectable genital fluid or saliva HIV RNA. Despite these limitations, further insight into the relationship of viral levels between blood, saliva and genital fluids of men and women was achieved using the A5077 clinical trial design.

In summary, our results highlight that plasma HIV RNA may not always reflect genital tract HIV shedding or resistance patterns, with differences in detected resistance in a minority of subjects. Importantly, a subset of treatment-experienced men and women continue to have genital tract HIV shedding implying continued risk of HIV transmission and potentially transmission of drug resistant virus. Further investigation is needed to determine possible mechanisms for these differences [61], particularly with newer ART regimens that were not available at the time of our study. For example, these mechanisms may be sex specific and related to specific genital tract hormonal and other environment influences including the genital tract microbiome. Although assessment of plasma genotyping in blood can provide accurate surrogate information about drug resistance in genital-secretions, some discordances do occur bi-directionally between the blood and genital compartments, potentially due to tissue site-specific replication, providing insight into the mechanism of compartmentalization of HIV. In contradistinction to our study hypothesis, there was predominately resistance concordance between the blood and genital tract for both men and women. Nevertheless, further understanding of resistance discordance may aid in treatment and prevention of HIV transmission. The prevalence of drug resistant virus in genital-secretions will pose a significant challenge to pre-exposure prophylaxis as well as treatment as prevention strategies.

## Supporting Information

Protocol S1 **ACTG A5077 protocol: Virologic Studies in Compartmental Samples from HIV-infected Subjects.**
(DOC)
