# Supplementary material for: HIV-1 RNA Levels and Antiretroviral Drug Resistance in Blood and Non-Blood Compartments from HIV-1–Infected Men and Women enrolled in AIDS Clinical Trials Group Study A5077
Source: PLoS One. 2014 Apr 3;9(4):e93537. doi: 10.1371/journal.pone.0093537 (PMC3974754; doi:10.1371/journal.pone.0093537)
Supplement: Protocol S1 — ACTG A5077 protocol: Virologic Studies in Compartmental Samples from HIV-infected Subjects. (DOC) [file pone.0093537.s001.doc]

# A5077

**VIROLOGIC STUDIES IN COMPARTMENTAL SAMPLES FROM**

**HIV-INFECTED SUBJECTS CHANGING OR INITIATING**

**POTENT ANTIRETROVIRAL THERAPY**

**A Multicenter Trial of the Adult AIDS Clinical Trials Group (AACTG)**

**Sponsored by:**

**The National Institute of Allergy and Infectious Diseases (NIAID)**

**The AACTG HIV Disease RAC Chair: Scott Hammer, M.D.**

**Protocol Chair: Alejo Erice, M.D.**

**Protocol Vice Chairs: Robert W. Coombs, M.D., Ph.D.**

**Susan A. Fiscus, Ph.D.**

**Clinical Trials Specialist: Kellye Maxwell, M.S.**

**FINAL Version 1.0**

**11/02/00**

Protocol Chair

Alejo Erice, M.D.

Department of Laboratory

Medicine and Pathology

Department of Medicine,

Infectious Disease Division

University of Minnesota

Box 437 Mayo

420 Delaware Street, S.E.

Minneapolis, MN 55455

Phone: (612) 626-0920

FAX: (612) 625-5468

E-mail: [erice001@tc.umn.edu](mailto:ERICE001@TC.UMN.EDU)

Protocol Vice Chair and Virologist

Robert W. Coombs, M.D., Ph.D.

Department of Laboratory Medicine

Division of Virology, Box 359690

University of Washington

Harborview Medical Center

Research & Training Building, 706C

325 9^th^ Avenue

Seattle, WA 98104-2499

Phone:(206) 341-5201

FAX: (206) 341-5203

E-mail: [bcoombs@u.washington.edu](mailto:BCOOMBS@U.WASHINGTON.EDU)

Protocol Vice Chair

Susan A. Fiscus, Ph.D.

Department of Microbiology & Immunology

University of North Carolina at Chapel Hill

School of Medicine

Mary Ellen Jones Building, Room 709

CB-7140

Chapel Hill, NC 27599-7140

Phone: (919) 966-6872

FAX: (919) 966-9873

E-mail: fiscussa@med.unc.edu

Clinical Trials Specialist

Kellye Maxwell, M.S.

AACTG Operations Center

6101 Executive Boulevard, Suite 350

Rockville, MD 20852

Phone: (301) 230-3150 ext. 189

FAX: (301) 816-0938

E-mail: kmaxwell@s-3.com

Protocol Statistician

Ronald J. Bosch, Ph.D.

HIV Disease, Adult Division,

Statistical and Data Analysis Center

Harvard School of Public Health

FXB Building, Room 603

651 Huntington Avenue,

Boston, MA 02115-6017

Phone: (617) 432-3024

FAX: (617) 432-2843

E-mail: [ronbosch@sdac.harvard.edu](mailto:RONBOSCH@SDAC.HARVARD.EDU)

Protocol Data Manager

Sandra Oyola, M.T.(ASCP)

Frontier Science and Technology Research

Foundation, Inc.

4033 Maple Road

Amherst, NY 14226-1056

Phone: (716) 834-0900 X 293

FAX: (716) 834-8675

E-mail: [oyola.sandra@fstrf.org](mailto:oyola.sandra@fstrf.org)

Field Representative

Mary Shoemaker, R.N.

University of Rochester Medical Center

601 Elmwood Avenue

P.O. Box 689

Rochester, NY 14642

Phone: (716) 275-4768

FAX: (716) 273-1055

E-mail: mary_shoemaker@urmc.rochester.edu

Investigators/WHC Representatives

Susan Cu-Uvin, M.D.

The Miriam Hospital, Brown University

164 Summit Avenue

Providence, RI 02906

Phone: (401) 793-4775

FAX: (401) 455-3485

E-mail: [susan_uvin@brown.edu](mailto:susan_uvin@brown.edu)

Amneris E. Luque, M.D.

University of Rochester Medical Center

School of Medicine & Dentistry

Room 3-6209

601 Elmwood Avenue

P. O. Box 689

Rochester, NY 14642-0001

Phone: (716) 275-5871

FAX: (716) 442-9328

E-mail: Amneris_Luque@URMC.Rochester.edu

CCG Representative

Vernella French

Tulane-LSU CAB

13963 Explorers Avenue

New Orleans, LA 70129

Phone: (504) 255-6325

E-mail: [vernella.french@usda.gov](mailto:vernella.french@usda.gov)

RAC Representative

David Katzenstein, M.D.

Division of Infectious Disease, S156

Stanford University Medical Center

300 Pasteur Drive

Stanford, CA 94305-5107

Phone: (650) 725-8304

FAX: (650) 725-2395

E-mail: [davidkk@leland.stanford.edu](mailto:DAVIDKK@LELAND.STANFORD.EDU)

Laboratory Technologist

Estelle M. Piwowar-Manning, M.T. (ASCP)

Department of Pathology

Johns Hopkins University Hospital

Pathology Room 313

600 North Wolfe Street

Baltimore, MD 21287

Phone: (410) 614-6736

FAX: (410) 614-0430

E-mail: epiwowa@jhmi.edu

#### Laboratory Data Coordinator

Mary Jo Werder, B.S.

Frontier Science and Technology

Research Foundation, Inc.

4033 Maple Road

Amherst, NY 14226-1056

Phone: (716) 834-0900 ext. 282

Fax: (716) 834-8432

E-mail: [Werder.maryjo@fstrf.org](mailto:Werder.maryjo@fstrf.org)

PARTICIPATING SITES

A5077 is open to all interested Adult ACTUs and their subunits.

STUDY MANAGEMENT [7](#__RefHeading___Toc497903665)

SCHEMA [9](#__RefHeading___Toc497903666)

1.0 INTRODUCTION [11](#__RefHeading___Toc497903667)

1.1 Background and Rationale [11](#__RefHeading___Toc497903668)

1.2 Study Design [14](#__RefHeading___Toc497903669)

2.0 STUDY OBJECTIVES [16](#__RefHeading___Toc497903670)

2.1 Primary [16](#__RefHeading___Toc497903671)

2.2 Secondary [16](#__RefHeading___Toc497903672)

3.0 SELECTION AND ENROLLMENT OF SUBJECTS [16](#__RefHeading___Toc497903673)

3.1 Inclusion Criteria [16](#__RefHeading___Toc497903674)

3.2 Exclusion Criteria [18](#__RefHeading___Toc497903675)

3.3 Enrollment Procedures [18](#__RefHeading___Toc497903676)

3.4 Coenrollment Guidelines [19](#__RefHeading___Toc497903677)

4.0 CLINICAL AND LABORATORY EVALUATIONS [20](#__RefHeading___Toc497903678)

4.1 Screening/Eligibility Evaluations [20](#__RefHeading___Toc497903679)

4.2 Preentry Evaluations [21](#__RefHeading___Toc497903680)

4.3 Entry Evaluations [21](#__RefHeading___Toc497903681)

4.4 On Study Evaluations [23](#__RefHeading___Toc497903682)

4.5 Virologic Failure Evaluations [25](#__RefHeading___Toc497903683)

4.6 Premature Study Discontinuation [26](#__RefHeading___Toc497903684)

5.0 DATA COLLECTION AND MONITORING AND ADVERSE EXPERIENCE REPORTING [26](#__RefHeading___Toc497903685)

5.1 Records to Be Kept [26](#__RefHeading___Toc497903686)

5.2 Role of Data Management [26](#__RefHeading___Toc497903687)

5.3 Regional Monitoring [27](#__RefHeading___Toc497903688)

6.0 STUDY TREATMENT [27](#__RefHeading___Toc497903689)

6.1 Criteria for Permanent Study Discontinuation [27](#__RefHeading___Toc497903690)

7.0 STATISTICAL CONSIDERATIONS [28](#__RefHeading___Toc497903691)

7.1 General Design Issues [28](#__RefHeading___Toc497903692)

7.2 Endpoints [28](#__RefHeading___Toc497903693)

7.3 Sample Size and Accrual [29](#__RefHeading___Toc497903694)

7.4 Monitoring Plan [33](#__RefHeading___Toc497903695)

7.5 Analysis Plan [33](#__RefHeading___Toc497903696)

8.0 HUMAN SUBJECTS [35](#__RefHeading___Toc497903697)

8.1 Institutional Review Board (IRB) Review and Informed Consent [35](#__RefHeading___Toc497903698)

8.2 Subject Confidentiality [35](#__RefHeading___Toc497903699)

8.3 Study Discontinuation [35](#__RefHeading___Toc497903700)

9.0 PUBLICATION OF RESEARCH FINDINGS [35](#__RefHeading___Toc497903701)

10.0 BIOHAZARD CONTAINMENT [35](#__RefHeading___Toc497903702)

11.0 REFERENCES [37](#__RefHeading___Toc497903703)

APPENDICES

I. SCHEDULE OF EVENTS

II. BLOOD COLLECTION, PROCESSING, STORAGE, AND SHIPMENT PROCEDURES

III. SALIVA Collection, Processing, STORAGE, AND SHIPMENT PROCEDURES

IV. GENITAL SECRETIONS COLLECTION, PROCESSING, STORAGE, AND SHIPMENT PROCEDURES

V. LYMPHOID TISSUE (LT) COLLECTION, PROCESSING, AND SHIPPING PROCEDURES

VI. SAMPLE INFORMED CONSENT

###### STUDY MANAGEMENT

All questions concerning this protocol should be sent via e-mail to ACTG.TEAMA5077@fstrf.org. The appropriate team member will respond to your questions via e-mail with a “cc” to ACTG.TEAMA5077@fstrf.org. A response should generally be received within 24 hours (Monday - Friday).

Sites registering to this study should contact the Computer Support Group at the Data Management Center via e-mail ([actg.user.support@fstrf.org](mailto:actg.user.support@fstrf.org)) to have the relevant personnel at the site added to the ACTG.PROTA5077 e-mail group as soon as possible. Inclusion in the protocol e-mail group will ensure that sites receive important information about the study during its implementation and conduct.

**FOR CLINICAL MEDICAL MANAGEMENT AND COENROLLMENT, THE PROTOCOL CHAIR OR VICE CHAIR(S) WILL RESPOND:**

- Send an e-mail message to ACTG.TEAMA5077@fstrf.org (ATTN: Alejo Erice, M.D., Robert Coombs, M.D., Ph.D., and Susan Fiscus, Ph.D.).
- Include the PID.
- Give a brief relevant history.
- Give the date of expected randomization, if applicable.

**FOR QUESTIONS SPECIFICALLY RELATED TO IMMUNOLOGIC OR VIROLOGIC LABORATORY TESTS, THE PROTOCOL VIROLOGIST WILL RESPOND:**

- Send an e-mail message to ACTG.TEAMA5077@fstrf.org (ATTN: Robert Coombs, M.D., Ph.D.).

**FOR QUESTIONS ABOUT INCLUSION/EXCLUSION CRITERIA, THE SCHEDULE OF EVENTS, CASE REPORT FORMS, TRANSFERS, DELINQUENCIES, AND OTHER DATA MANAGEMENT ISSUES, THE DATA MANAGER WILL RESPOND:**

- Send an e-mail message to ACTG.TEAMA5077@fstrf.org (ATTN: Sandra Oyola, M.T.(ASCP)).
- Include the PID.
- Give a detailed description of the question.

STUDY MANAGEMENT (Cont.)

**FOR RANDOMIZATION QUESTIONS OR PROBLEMS, THE SDAC/DMC PROGRAMMERS WILL RESPOND:**

- Call the SDAC/DMC Randomization Desk at (716) 834-0900, x301.

**FOR COMPUTER AND SCREEN PROBLEMS, THE SDAC/DMC PROGRAMMERS WILL RESPOND:**

- Send an e-mail message to ACTG.USER.SUPPORT.

**FOR PROTOCOL QUESTIONS, THE CLINICAL TRIALS SPECIALIST WILL RESPOND:**

- Send an e-mail message to ACTG.TEAMA5077@fstrf.org (ATTN: Kellye Maxwell, M.S.).

**TO REQUEST COPIES OF THE PROTOCOL:**

- Hard copies: Send an e-mail message to ACTG.OPS (ATTN: Diane Delgado).
- Electronic copies can be downloaded from the Members area of the AACTG Web site (http://aactg.s-3.com).

**FOR SITE REGISTRATION:**

- Send an e-mail message to ACTG.SITEREG.

**FOR AER QUESTIONS:**

- Send an e-mail message to ACTG.ADR, or
- Call 1-800-537-9979.

Any phone calls must be documented by e-mail to ACTG.TEAMA5077@fstrf.org. This will be the site’s responsibility.

###### SCHEMA

VIROLOGIC STUDIES IN COMPARTMENTAL SAMPLES

FROM HIV-INFECTED SUBJECTS CHANGING OR INITIATING

POTENT ANTIRETROVIRAL THERAPY

DESIGN: A multicenter, cross-sectional, and longitudinal study designed to characterize differences in viral load and virus genotype and to assess the association between viral load and viral characteristics in blood (plasma and PBMCs) and nonblood compartments (saliva, genital secretions, and lymphoid tissues).

SAMPLE SIZE: 164 subjects.

POPULATION: • HIV-1-infected male and female subjects > 13 years of age initiating or changing potent antiretroviral therapy.

• Plasma HIV-1 RNA > 2000 copies/mL.

• Willing to contribute samples of blood, saliva, and either genital secretions or lymph tissues, or both.

NOTE: No antiretroviral drugs will be provided by A5077.

NOTE: Potent antiretroviral therapy is defined as a minimum of three antiretroviral drugs that includes any of the following combinations:

- two nucleoside analogues and a non-nucleoside reverse transcriptase inhibitor (NNRTI);
- two nucleoside analogues and abacavir;
- two nucleoside analogues and a protease inhibitor;
- two protease inhibitors and an NNRTI;
- two protease inhibitors and a nucleoside analogue; or
- a nucleoside analogue, an NNRTI, and a protease inhibitor.

STRATIFICATION: Subjects will be stratified by sex to ensure equal (50%) enrollment of males (n = 82) and females (n = 82).

DURATION: Subjects will participate in this study for 96 weeks.

SCHEMA, (Cont.)

OBJECTIVES:

Primary: 1. Evaluate the relationship between baseline viral load in blood and in nonblood compartments and the time to virologic failure.

2. Evaluate the relationship between viral load in nonblood compartments at the time of suppression of plasma HIV-1 RNA levels and the time to subsequent virologic failure.

3. Analyze the frequency of detecting HIV-1 in saliva, genital secretions, and lymphoid tissues from subjects with persistently suppressed plasma HIV-1 RNA levels.

Secondary: 1. Investigate whether loss of HIV-1 suppression occurs earlier in blood or in nonblood compartments in subjects who have previously achieved viral suppression in blood and nonblood compartments.

2. Investigate whether HIV-1-drug-resistant mutations not present at baseline are acquired earlier in blood or in nonblood compartments.

# INTRODUCTION

## Background and Rationale

Official guidelines for the treatment of HIV-1 infection indicate that the goal of antiretroviral therapy is maximal suppression of HIV-1 replication (1). The advent of potent antiretroviral therapy has made it possible to suppress HIV-1 replication to an extent that the virus is no longer detectable in the plasma of infected individuals (1). However, recent studies have demonstrated the presence of a latent pool of replication-competent virus in memory CD4+ lymphocytes of HIV-1-infected individuals (2, 3). Initial observations suggested that this viral reservoir was very stable and did not evolve over time in individuals with suppressed HIV-1 replication (2). More recently, studies involving a limited number of subjects have demonstrated that there is ongoing replication of latent virus in the blood and lymphoid tissues of some subjects receiving potent antiretroviral therapy who have had suppressed plasma HIV-1 RNA concentrations for prolonged periods of time (4-6). This continued viral persistence and replication could eventually result in virologic failure and clinical progression, selection, and transmission of resistant HIV-1.

Because of the continued viral replication in cellular compartments and tissues in subjects with suppressed plasma HIV-1 RNA concentration, it is possible to hypothesize that the timing of virologic events differs in different body compartments, and that from a virologic standpoint, treated individuals with suppressed plasma HIV-1 RNA levels do not constitute a homogeneous group. Virologic studies in compartments other than blood (lymphoid tissues, genital secretions, saliva) in HIV-1-infected individuals prior to and while receiving potent antiretroviral therapy would help to identify, quantify, and determine the significance of these viral reservoirs, and to determine the long-term effect of antiretroviral therapies in virus present in these compartments.

A5077 is designed to analyze relationships between viral load in blood and nonblood compartments and time to virologic failure in subjects initiating or changing potent antiretroviral therapy. The virologic analyses in blood and nonblood compartments that are planned as part of this protocol might help to better segregate subjects with suppressed plasma HIV-1 RNA levels and identify those subjects who will subsequently develop virologic failure. Of particular interest are cross-sectional and longitudinal analyses of blood and nonblood compartmental samples obtained from subjects at the following time points: 1) at study entry (baseline); 2) at the time of virologic suppression in plasma (< 200 HIV-1 RNA copies/mL); 3) at the time of virologic failure (HIV-1 RNA levels > 200 copies/mL); and 4) during follow-up. Characterization of the viral burden (HIV-1 RNA, proviral DNA) and other characteristics of the virus (genotypic resistance patterns, viral evolution, synctium-inducing/nonsynctium-inducing (SI/NSI), fitness, etc.) in blood and in specimens from nonblood compartments collected at these time points will contribute to the understanding of the virologic factors influencing the long-term effects of potent antiretroviral therapy.

A5077 includes sampling of lymphoid tissues from study participants because lymphoid organs constitute a major HIV-1 reservoir and are primary sites for HIV-1 replication (7,8). By using in situ hybridization and other quantitative methodologies, it has been possible to characterize and quantitate the different pools of HIV-1 in the lymphoid organs of infected individuals (7-9). Quantitative virologic studies in lymphoid tissue specimens obtained from antiretroviral-naïve individuals have shown that potent antiretroviral therapy that reduces plasma HIV-1 RNA to undetectable levels is also associated with significant reductions in the amount of HIV-1 in lymphoid tissues (10,11). These reductions in tissue viral burden are the consequence of a decline in the follicular dendritic cell (FDC)-associated virus together with a reduction of HIV-1 mRNA expression and proviral HIV-1 DNA in tissue CD4+ mononuclear cells. Despite these reductions, HIV-1 is still present in lymphoid tissues of individuals with suppressed plasma HIV-1 RNA levels (10).

In addition to these residual viral pools, recent studies suggest that there is ongoing HIV-1 replication in the lymphoid tissues of individuals with undetectable plasma HIV-1 RNA levels during potent antiretroviral therapy (4). Because viral replication under selective antiretroviral pressure could select for drug-resistant HIV-1 (12), it is possible for resistant virus to emerge in the lymphoid tissue compartments of individuals on potent antiretroviral therapy who have undetectable plasma HIV-1 RNA levels. Characterization of HIV-1 sequences in peripheral blood leukocytes in a small number of these individuals suggests that in these cases HIV-1 replication is associated with the evolution of viral sequences but not with the emergence of virus containing drug-resistant mutations in the reverse transcriptase and/or the protease regions, at least in the peripheral blood mononuclear cells (PBMCs) (4). Whether this is also true in the lymphoid organs of individuals receiving potent antiretroviral therapy with suppressed plasma HIV-1 RNA levels requires studying lymphoid tissue specimens collected prospectively from these subjects.

In contrast, analysis of HIV-1 sequences in lymphoid tissues from patients with persistently detectable plasma HIV-1 RNA levels while receiving potent antiretroviral therapy has established that viral levels are higher in tissues as compared with plasma and that resistance mutations accumulate in this compartment (13,14). Whether the appearance of these resistant viruses in lymphoid tissues precedes or occurs simultaneously with the appearance of resistant virus in the peripheral blood also requires studying lymphoid tissue specimens collected prospectively from patients receiving potent antiretroviral therapy.

Because of the importance of the genital tract in the transmission of HIV-1, A5077 includes sampling of genital secretions from study participants for virologic studies. Relatively few studies have been conducted investigating HIV-1 expression, viral evolution, and the effects of potent antiretroviral therapy in this compartment. Data from several cross-sectional studies suggest that 60%-75% of men shed HIV-1 RNA in the seminal plasma and that 65%-80% have detectable HIV-1 DNA in seminal cell pellets (15-18). Similar studies in women demonstrate considerably more variation, with 30%-88% of women having detectable HIV-1 RNA in the genital tract, and 40%-70% having detectable HIV-1 DNA in this compartment (19-23). Most of this variation is probably attributable to differences in the method used for sample collection (vaginal swab versus cervical swab versus cervicovaginal lavage), as well as to differences in the assays used to determine viral load (commercial versus in-house assay) (24,25). However, additional variation in viral HIV-1 RNA levels may arise from the hormonal effects of menses on viral shedding from the endocervical canal (26). Despite these differences, the overall conclusions from these studies have been quite similar. HIV-1 levels in the genital tract are generally lower than those observed in the blood at the same time, but still correlate significantly with HIV-1 RNA levels in plasma. However, there are a few individuals who have an HIV-1 viral load level in the genital tract that is considerably greater than their viral load level in plasma (22,27).

Longitudinal studies involving subjects initiating potent antiretroviral therapy have demonstrated that the decrease of the viral load in the genital tract parallels that in the blood (17,22,28,29). In a small study of subjects with plasma HIV-1 RNA levels < 400 copies/mL for at least 2 years, HIV-1 RNA was present in 1/21 seminal plasma samples and 2/2 cervical specimens (30). In a larger study of 114 men with similar plasma HIV-1 RNA levels, only 2 (2%) had detectable HIV-1 RNA in seminal plasma, and 16% had detectable HIV-1 DNA in seminal cells (31). Using more sensitive techniques, proviral HIV-1 DNA was detected in the seminal cells of 4 of 7 men with long-term suppression of HIV-1 in blood plasma, and HIV-1 was isolated from the seminal cells of two of the men (32). In contrast

to genital tract HIV-1 RNA, detection of HIV-1 DNA in genital secretions does not appear to correlate well with plasma HIV-1 RNA levels nor does it seem to be affected by antiretroviral therapy (21,31).

Incomplete suppression of HIV-1 replication in the genital tract could lead to the development of antiretroviral drug resistance, and potentially to transmission of drug-resistant HIV-1. Several studies have documented the presence of drug-resistant HIV-1 in genital secretions from men and women (33-35). In addition, sexual transmission of HIV-1 variants with mutations conferring resistance to reverse transcriptase and/or protease inhibitors has been reported (36-40). Whether resistant HIV-1 emerges in the genital tract before, simultaneously, or after its appearance in the peripheral blood is not known at the present time.

Much less is known about the oral cavity in terms of its capacity to serve as a reservoir for latent virus and as a vehicle for transmission of HIV-1. HIV-1 RNA and DNA have been detected in cell-free or whole saliva (41-44). Similar to the genital tract, HIV-1 RNA levels in saliva are generally lower than those in plasma, although a few subjects have higher viral loads when compared with those in blood (Shugars, personal communication). Since it bathes the oral lymph glands, it is possible that the saliva may serve as a surrogate specimen from which to study virologic events occurring in the lymphoid tissues. Collecting saliva is simple, inexpensive, and not associated with the risks of surgical procedures. To date, the presence of HIV-1 containing drug-resistant mutations has not been assessed in saliva.

The methodologies for sampling, processing, storing, and assaying nonblood compartment samples for virologic studies are not standardized. A5077 provides a uniform approach to obtain adequate numbers of compartmental samples from HIV-1-infected individuals that are representative of different HIV-1 disease stages and/or different potent antiretroviral therapy regimens. Analysis of these samples using state-of-the-art methods would contribute to the understanding of virologic determinants of response to potent antiretroviral therapy.

## Study Design

This study is designed to provide access to compartmental samples for cross-sectional and long-term longitudinal analyses. For the purpose of this study, compartments refer to blood, saliva, genital secretions, and lymphoid tissues.

A total of 164 subjects > 13 years of age will be enrolled in this study. Accrual will be monitored to ensure equal (50%) enrollment of males (n = 82) and females (n = 82). Enrollment will consist of subjects who are changing or initiating potent antiretroviral therapy. No antiretroviral drugs will be provided by A5077.

NOTE: Potent antiretroviral therapy is defined as a minimum of three antiretroviral drugs that includes any of the following combinations: two nucleoside analogues and a non-nucleoside reverse transcriptase inhibitor (NNRTI); two nucleoside analogues and abacavir; two nucleoside analogues and a protease inhibitor; two protease inhibitors and an NNRTI; two protease inhibitors and a nucleoside analogue; or a nucleoside analogue, an NNRTI, and a protease inhibitor.

Subjects will be required to contribute samples of blood at screening, preentry, study entry (prior to changing or initiating potent antiretroviral therapy), week 8 and every 8 weeks thereafter until week 96, within 30 days of confirmed virologic failure, and at the time of premature study discontinuation. Subjects will be required to contribute samples of saliva and either genital secretions or lymphoid tissue, or both, at study entry (prior to changing or initiating potent antiretroviral therapy), weeks 16, 48, and 96, within 30 days of confirmed virologic failure, and at the time of premature study discontinuation. Please note that if total blood volumes exceed safe limits (the American Red Cross has a limit of 450 mL of blood over 56 days for healthy volunteers), blood samples will not be drawn for A5077. In these instances, the A5077 protocol team will request access to plasma and PBMC samples from the coenrolled study(ies) (if applicable).

For the purpose of this study, suspected virologic failure is defined as the first unconfirmed HIV-1 RNA level > 200 copies/mL > 16 weeks after changing or initiating potent antiretroviral therapy, and virologic failure is defined by confirmed plasma HIV-1 RNA levels > 200 copies/mL > 16 weeks after changing or initiating potent antiretroviral therapy. Subjects who experience confirmed virologic failure should have the virologic failure evaluations performed within 30 days (see section 4.52). After subjects have the virologic failure evaluations performed, they should remain on study and resume their regularly scheduled study visits until week 96. Subjects who change their antiretroviral drug regimens during their participation in A5077 should also continue on study and have the required protocol evaluations performed until their week 96 visit.

Because nonblood compartment specimens are more difficult to obtain than blood specimens, the current primary objectives have been designed to complement

each other and to optimally use nonblood compartment data that will be obtained as part of the study.

# STUDY OBJECTIVES

## Primary

2.11 Evaluate the relationship between baseline viral load in blood and in nonblood compartments and the time to virologic failure.

2.12 Evaluate the relationship between viral load in nonblood compartments at the time of suppression of plasma HIV-1 RNA levels and the time to subsequent virologic failure.

2.13 Analyze the frequency of detecting HIV-1 in saliva, genital secretions, and lymphoid tissues from subjects with persistently suppressed plasma HIV-1 RNA levels.

## Secondary

2.21 Investigate whether loss of HIV-1 suppression occurs earlier in blood or in nonblood compartments in subjects who have previously achieved viral suppression in blood and nonblood compartments.

2.22 Investigate whether HIV-1-drug-resistant mutations not present at baseline are acquired earlier in blood or in nonblood compartments.

# SELECTION AND ENROLLMENT OF SUBJECTS

## Inclusion Criteria

3.11 Ability and willingness to give signed written informed consent.

NOTE: Subjects < 18 years of age must have the signed written informed consent of a parent or legal guardian.

3.12 Documentation of HIV-1 infection by any licensed ELISA test kit and confirmed by either Western blot, HIV-1 culture, HIV-1 antigen, plasma HIV-1 RNA, or a second antibody test by a method other than ELISA at any time prior to study entry.

3.13 Changing or initiating potent antiretroviral therapy.

NOTE: Potent antiretroviral therapy is defined as a minimum of three antiretroviral drugs that includes any of the following combinations: two nucleoside analogues and a non-nucleoside reverse transcriptase inhibitor (NNRTI); two nucleoside analogues and abacavir; two nucleoside analogues and a protease inhibitor; two protease inhibitors and an NNRTI; two protease inhibitors and a nucleoside analogue; or a nucleoside analogue, an NNRTI, and a protease inhibitor.

NOTE: No antiretroviral drugs will be provided by A5077.

3.14 Documentation of plasma HIV-1 RNA PCR or bDNA value > 2000 copies/mL by any of the following certified methods within 60 days of study entry:

3.141 Chiron bDNA version 3.0 (lower limit of detection, 50 copies/mL). Other versions of the Chiron assay are not acceptable.

3.142 Standard Roche Amplicor HIV-1 Monitor™ assay (lower limit of detection, 400 copies/mL).

3.143 UltraSensitive Roche Amplicor HIV-1 Monitor™ assay (lower limit of detection, 50 copies/mL).

3.144 Organon Teknika Nucleic Acid Sequence-Based Amplification (NASBA)™ HIV-1 RNA QT assay (lower limit of detection, 400 copies/mL).

3.145 Organon Teknika NucliSens™ HIV-1 RNA QT assay (lower limit of detection, 40 copies/mL).

3.15 Males and females > 13 years.

3.16 Willingness to contribute samples of blood, saliva, and either genital secretions or lymphoid tissue, or both, at the required study visits.

3.17 Willingness to allow the protocol team to have access to and use of data derived from clinic records and other coenrolled studies (if applicable) while participating in A5077.

3.18 Willingness to allow the protocol team to access results of blood tests and/or use blood samples from other coenrolled studies (if applicable) or clinic visits while participating in A5077.

3.19 Negative urine or serum ß-HCG pregnancy test (women with reproductive potential only) within 14 days of study entry.

NOTE: If a woman becomes pregnant while participating in A5077, she will be allowed to continue on study, but only allowed to contribute blood and saliva samples until the pregnancy is completed. Following completion of the pregnancy, she will be allowed to resume genital secretion and/or lymphoid tissue collection procedures.

3.110 Subjects not enrolled in other AACTG clinical trials must receive their primary HIV-related care at an AACTG site.

## Exclusion Criteria

3.21 Any active opportunistic infections (OIs), opportunistic diseases, intercurrent illnesses, or other infections, including but not limited to active lower genital tract infections, requiring a new or a change in medication < 14 days prior to study entry.

NOTE: Subjects who require a new or a change in medication for oral candidiasis or oral hairy leukoplakia < 14 days prior to study entry are not excluded from participation.

3.22 Any use of immunomodulatory agents < 14 days prior to study entry.

3.23 Active immunization < 14 days prior to study entry.

## Enrollment Procedures

3.31 Prior to implementation, sites must have the protocol approved by their local institutional review board (IRB) and be registered with the Regulatory Operations Center Site Registration Desk. Site registration must occur before any subjects can be enrolled.

3.32 The subjects will first be assessed for admission to the study. Once it has been determined that the subject meets the study entry criteria, details of the study will be carefully discussed with the subject. The subject (or parent or legal guardian if the subject is younger than 18 years of age) will be asked to read and sign a consent form approved by the ACTU’s IRB.

3.33 The Division of AIDS has concluded that this protocol does NOT meet Federal requirements governing prisoner participation in clinical trials and should NOT be considered by local IRBs for the recruitment of prisoners.

## Coenrollment Guidelines

3.41 Coenrollment Guidelines for AACTG Studies

If eligible, the team strongly encourages sites to coenroll subjects into the following studies:

A5001 (ALLRT)

ACTG 736

A5076

A5029

Permission to enroll in any other AACTG and non-AACTG clinical trials is required from the A5077 protocol chair or vice chair(s). Subjects should not be coenrolled in studies where the combined total blood volumes collected will exceed appropriate and safe limits. The American Red Cross has a limit of 450 mL of blood over 56 days for healthy volunteers.

3.42 Guidelines for Enrolling Non-AACTG Subjects

Subjects not enrolled in other AACTG clinical trials must receive their primary HIV-related care at an AACTG site.

# CLINICAL AND LABORATORY EVALUATIONS

All HIV-related diagnoses and AIDS-defining events that occur prior to study entry and during the study must be recorded in source documentation and on the CRFs and entered into the database. All anti-HIV medications, immune-based therapies, and HIV vaccines the subject has ever taken prior to study entry and during the study must be recorded in source documentation and on the CRFs and entered into the database. Signs and symptoms, regardless of grade, must be recorded in source documentation only.

If CD4/CD8 cell counts or HIV-1 RNA levels are measured as part of a coenrolled study at the same time as an A5077 protocol visit, or total blood volumes exceed safe limits, they do not need to be repeated for A5077. If available, the results of the test(s) should be obtained and entered onto the appropriate A5077 CRF. Please note that hematology (complete blood count [CBC] including differential) is only required if CD4/CD8 cell counts are measured as part of an A5077 visit.

Each subject’s CD4/CD8 and plasma HIV-1 RNA evaluations must be performed by an ACTG-certified laboratory throughout the study except at screening. Because of the diurnal variation in CD4 cell counts, CD4 determinations for each subject should be obtained consistently in either the morning or the afternoon throughout the study. Preentry and entry HIV-1 RNA levels and CD4/CD8 cell counts will be averaged to determine the baseline values for each.

Please note that all of the compartmental assessments (saliva, genital secretions, and lymphoid tissues) and some of the blood assessments performed as part of A5077 will

not be performed in real time. These samples will be stored and batch-tested at a later date. Subjects who have their CD4/CD8 cell counts and HIV-1 RNA levels measured as part of the A5077 protocol evaluations will be provided with real time results.

## Screening/Eligibility Evaluations

All screening evaluations must be performed within 30 days of study entry unless otherwise noted.

4.11 Details of the study should be carefully discussed with the subject and the subject (or parent or legal guardian if the subject is younger than 18 years of age) asked to read and sign a consent form approved by a local IRB prior to implementation of any study-related evaluations.

4.12 Documentation of HIV-1 infection anytime prior to study entry.

4.13 Whole blood for plasma HIV-1 RNA measurement or documentation of plasma HIV-1 RNA PCR or bDNA value > 2000 copies/mL within 60 days of study entry.

4.14 Complete clinical assessment to include a history of all HIV-related diagnoses and AIDS-defining events, and a signs and symptoms assessment with symptom-directed physical exam. If any signs or symptoms of a genitourinary tract infection are noted, the subject should be evaluated and treated for at least 14 days and the infection considered resolved prior to entry.

4.15 Medication history to include all anti-HIV medications, immune-based therapies, and HIV vaccines ever taken prior to study entry.

4.16 Women of reproductive potential must have a negative urine or serum β-HCG pregnancy test within 14 days prior to study entry.

## Preentry Evaluations

All pre-entry evaluations must be performed within 30 days of study entry unless otherwise noted. Preentry and entry evaluations should be separated by at least 24 hours.

4.21 Hematology: CBC with differential.

4.22 CD4 and CD8 cell counts (absolute and percentage).

4.23 Plasma HIV-1 RNA (using the UltraSensitive Roche assay; see Appendix II).

## Entry Evaluations

Entry specimens must be obtained prior to changing or initiating potent antiretroviral therapy. Study entry evaluations should be performed within 30 days of the screening evaluations. Preentry and entry evaluations should be separated by at least 24 hours.

4.31 Noncompartmental Evaluations

4.311 Targeted physical exam to include signs/symptoms and new HIV-related diagnoses. If any signs or symptoms of a genitourinary tract infection are noted, the subject should be evaluated and entry delayed until the subject has been treated for at least 14 days and the infection is considered resolved.

4.312 Medication update to include changes in anti-HIV medications, immune-based therapies, and HIV vaccines.

4.313 Hematology: CBC with differential.

4.314 CD4 and CD8 cell counts (absolute and percentage).

4.315 Plasma HIV-1 RNA (using the UltraSensitive Roche assay; see Appendix II).

4.32 Compartmental Evaluations

4.321 Whole blood will be collected for storage of plasma and PBMC samples (see Appendix II). The following evaluations will be performed unless total blood volumes exceed safe limits (the American Red Cross has a limit of 450 mL of blood over 56 days for healthy volunteers):

• Plasma for HIV-1 genotypic resistance studies.

• PBMC storage for HIV-1 proviral DNA studies.

NOTE: If the subject is enrolled in other studies and blood volumes do not permit obtaining an additional blood sample for A5077, the A5077 protocol team will request access to plasma and PBMC samples from the coenrolled study(ies) (see Appendix II).

4.322 Saliva: Samples of saliva should be obtained, processed, stored, and shipped as outlined in Appendix III. The following evaluations will be performed:

• HIV-1 RNA quantitation.

• HIV-1 genotypic resistance studies.

• HIV-1 proviral DNA studies.

4.323 Genital Secretions and/or Lymphoid Tissues: Subjects have the option to contribute either genital secretions or lymphoid tissues, or both. Samples should be obtained, processed, stored, and shipped as outlined in Appendices IV and V. The following evaluations will be performed:

• HIV-1 RNA quantitation.

• HIV-1 genotypic resistance studies.

• HIV-1 proviral DNA studies.

## On Study Evaluations

Noncompartmental evaluations are required at week 8 and every 8 weeks thereafter until week 96, within 30 days of confirmed virologic failure (section 4.5), and at the time of premature study discontinuation (section 4.6). Compartmental evaluations are required at weeks 16, 48, 96, within 30 days of confirmed virologic failure (section 4.5), and at the time of premature study discontinuation (section 4.6).

4.41 Noncompartmental Evaluations:

4.411 Clinical assessment to include updated signs/symptoms and new HIV-related diagnoses.

NOTE: If any signs or symptoms of a genitourinary tract infection are noted, the subject should be evaluated and, if indicated, treated. Sampling of genital secretions should be delayed until the infection is resolved.

4.412 Medication update to include changes in anti-HIV medications, immune-based therapies, and HIV vaccines.

4.413 Hematology: CBC with differential.

4.414 CD4 and CD8 cell counts (absolute and percentage).

4.415 Plasma HIV-1 RNA (using the UltraSensitive Roche assay; see Appendix II).

4.42 Compartmental Evaluations

4.421 Whole blood will be collected for storage of plasma and PBMC samples (see Appendix II). The following evaluations will be performed unless total blood volumes exceed safe limits (the American Red Cross has a limit of 450 mL of blood over 56 days for healthy volunteers):

• Plasma for HIV-1 genotypic resistance studies.

• PBMC storage for HIV-1 proviral DNA studies.

NOTE: If the subject is enrolled in other studies and blood volumes do not permit obtaining an additional blood sample for A5077, the A5077 protocol team will request access to plasma and PBMC samples from the coenrolled study(ies) (see Appendix II).

4.422 Saliva: Samples of saliva should be obtained, processed, stored, and shipped as outlined in Appendix III. The following evaluations will be performed:

• HIV-1 RNA quantitation.

• HIV-1 genotypic resistance studies.

• HIV-1 proviral DNA studies.

4.423 Genital Secretions and/or Lymphoid Tissues: Subjects have the option to contribute either genital secretions or lymphoid tissues, or both. Samples should be obtained, processed, stored, and shipped as outlined in Appendices IV and V. The following evaluations will be performed:

• HIV-1 RNA quantitation.

• HIV-1 genotypic resistance studies.

• HIV-1 proviral DNA studies.

## Virologic Failure Evaluations

4.51 Criterion for Virologic Failure

Subjects with suspected virologic failure (defined as HIV-1 RNA level > 200 copies/mL > 16 weeks after initiating or changing potent antiretroviral therapy) will be required to return to the clinic to have a confirmatory blood plasma HIV-1 RNA performed within 30 days.

NOTE: Since any vaccination or acute illness may cause an increase in viral load, specimens should be collected at least 14 days after vaccination or acute illness whenever possible.

4.52 Virologic Failure Evaluations

Virologic failure evaluations must be performed within 30 days of confirmed virologic failure (i.e., within 30 days from the date of the confirmatory HIV-1 RNA). The evaluations required at the time of virologic failure are the same as those required at week 96 (see section 4.4).

After subjects have the virologic failure evaluations performed, they should remain on study and resume their regularly scheduled study visits until week 96. Subjects who change their antiretroviral drug regimens during their participation in A5077 should also continue on study and have the required protocol evaluations performed until their week 96 visit.

NOTE: Subjects should only have the virologic failure evaluations performed the first time virologic failure is confirmed during their participation in A5077. Subjects who experience confirmed virologic failure subsequently while still participating in A5077 should NOT have the virologic failure evaluations performed again.

## Premature Study Discontinuation

Subjects who discontinue the study before week 96 must have the evaluations required at week 96 performed within 30 days of premature discontinuation. If a study visit in which compartmental evaluations was performed within 30 days prior to premature study discontinuation, these evaluations do not need to be repeated. Please refer to section 6.1 for the criteria for study discontinuation.

# DATA COLLECTION AND MONITORING AND ADVERSE EXPERIENCE REPORTING

## Records to Be Kept

Case report forms (CRFs) will be provided for each subject. Subjects must not be identified by name on any study documents. Subjects will be identified by the Patient Identification Number (PID) and Study Identification Number (SID) provided by the ACTG Data Management Center upon (randomization/ registration).

All data on the CRF must be legibly recorded in black ink or typed. A correction should be made by striking through the incorrect entry with a single line and entering the correct information adjacent to it. The correction must be initialed and dated by the investigator or a designated qualified individual. Any requested information that is not obtained as specified in the protocol should have an explanation noted on the CRF and in the source document as to why the required information was not obtained.

## Role of Data Management

5.21 Instructions concerning the recording of study data on CRFs or the entry of data in the computerized database will be provided by the ACTG Data Management Center. Each ACTU is responsible for keying the data in a timely fashion.

5.22 It is the responsibility of the ACTG Data Management Center to assure the quality of computerized data for each ACTG study. This role extends from protocol development to generation of the final study databases.

## Regional Monitoring

5.31 Site monitors under contract to the National Institute of Allergy and Infectious Diseases (NIAID) will visit participating clinical sites to review the research records for accuracy, completeness, and legibility. The monitors also will inspect sites' regulatory files to ensure that regulatory requirements are being followed.

5.32 Site visits will be made at main units and the larger subunits at standard intervals and more frequently as directed by NIAID.

5.33 The investigator will make study documents (e.g., consent forms, CRFs) and pertinent hospital or clinic records readily available for inspection by the site monitors for confirmation of the study data.

# STUDY TREATMENT

Treatment will not be provided as part of this study.

## Criteria for Permanent Study Discontinuation

6.11 Completion of study.

6.12 The subject or legal guardian refuses further follow-up evaluations.

6.13 The investigator determines that further participation would be detrimental to the subject’s health or well-being.

6.14 The subject fails to comply with the study requirements so as to cause harm to self or seriously interfere with the validity of the study results.

# STATISTICAL CONSIDERATIONS

## General Design Issues

This exploratory study will enroll 82 men and 82 women changing or initiating potent antiretroviral therapy in order to provide cross-sectional and longitudinal analyses of compartmental samples. Blood plasma viral load will be assessed at preentry, entry, week 8 and then every 8 weeks thereafter until week 96, within 30 days of confirmed blood plasma virologic failure, and at the time of premature study discontinuation. Nonblood compartments (saliva and either genital secretions or lymph tissues, or both) will be sampled at study entry (prior to changing or initiating potent antiretroviral treatment); weeks 16, 48, and 96; within 30 days of confirmed blood plasma virologic failure; and at the time of premature study discontinuation. Accrual is anticipated to take approximately 3 years.

Characterization of the viral burden (HIV-1 RNA, proviral DNA) and other characteristics of the virus (genotypic resistance patterns, viral evolution, SI/NSI, fitness, etc.) in compartmental specimens collected at these time points will contribute to understanding of the virologic factors influencing the long-term effect of antiretroviral therapy. Since it is anticipated that the nonblood virologic measures may not be available for all subjects at all time points, the primary

objectives of this study are designed to complement one another and to make optimal use of the available compartmental data.

## Endpoints

7.21 Primary Endpoints

7.211 Virologic failure

For the first primary objective, virologic failure will be defined as confirmed blood plasma HIV-1 RNA > 200 copies/mL at week 16 or later. Subjects without blood plasma HIV-1 RNA at week 16 or later will be considered virologic failures at week 16; subjects without an available confirmatory blood plasma viral load will be considered virologic failures.

7.212 Conditional virologic failure

For the second primary objective, virologic failure will occur at the first of two successive time points (> week 16) where blood plasma HIV-1 RNA is > 200 copies/mL. Subjects without an available confirmatory blood plasma viral load will be considered virologic failures. If a subject does not have virologic failure, the subject will be considered censored at the last time point with a blood plasma HIV-1 RNA measure.

7.213 Virus Detection

The endpoint for the third primary objective is the ability to detect virus from saliva, genital secretions, and lymphoid tissues.

7.22 Secondary Endpoints

7.221 Virologic events

The endpoints for the secondary objectives are the times indicating the loss of virologic suppression in blood and nonblood compartments, and the times of the appearance of HIV-1-drug-resistant mutations not present at baseline in blood and nonblood compartments.

## Sample Size and Accrual

7.31 Virologic Failure

To minimize the assumptions needed to estimate power for the first primary objective, we consider virologic failure as a binary endpoint (yes/no) and model the probability of virologic failure as a function of baseline blood plasma viral load and a nonblood viral load measure. Blood viral load and nonblood viral load are considered to have a bivariate normal distribution with correlation = 0.4 (as observed in ACTG 347 between blood plasma and seminal plasma viral load). The nonblood viral load is dichotomized at its mean, while blood viral load is modeled as a continuous predictor on the log_10_ scale, with mean = 4.477 log_10_ copies/mL (30,000 copies/mL) and SD = 0.7 (as observed in ACTG 175).

Power is determined by logistic regression simulations with a two-sided alpha = 0.05, where the effect of the nonblood viral load is evaluated in a model that already contains blood viral load. The rate of virologic failure for a subject with mean levels of the viral load measures is assumed to be 30%. For each log_10_ higher baseline blood viral load, the odds ratio (OR) for virologic failure is assumed to be 3 (approximately the OR observed in ACTG 364).

With 164 subjects enrolled, it is anticipated that baseline blood and nonblood viral loads will be available for 130 subjects (i.e., a 21% adjustment is made for the possibility of unavailable baseline measures). Then, there is 82% power to detect OR = 3.5, comparing subjects with high nonblood viral loads versus low nonblood viral loads in terms of virologic failure. This corresponds to a probability of virologic failure of 44% for a subject with high nonblood viral load compared with a probability of 19% for a subject with low nonblood viral load, both subjects having similar blood viral loads.

Similarly, there is 72% power to detect an OR = 3; this corresponds to virologic failure probabilities of 43% versus 20% for subjects having high and low nonblood viral loads, respectively.

Note that these power statements can be considered conservative, in that higher power is expected if the nonblood viral load is modeled as a continuous variable or if time-to-event methods are used instead of the binary, logistic regression approach used for the power calculations (and more so if the virologic failure rate is higher).

7.32 Conditional Virologic Failure

It is assumed that 61% of the enrolled subjects will be on study, have a week 16 nonblood viral load measurement, have blood plasma HIV-1 RNA < 200 copies/mL at week 16, and have at least one subsequent blood plasma viral load measure, implying a sample size of 100 for the analysis of the second primary objective. The proportion of these subjects with detectable nonblood viral load is expected to depend on the compartment of interest and also on the assay method.

Kovacs et al. (45) reported that 26% of women with blood plasma HIV-1 RNA < 500 copies/mL had detectable HIV-1 in the genital tract. It was observed in ACTG 320/866 that out of 11 (9 at week 24 and 2 at week 40) paired on-treatment blood plasma and female genital tract specimens in which blood plasma HIV-1 RNA was undetectable (< 400 copies/mL by NucliSens), 9% (one) had detectable HIV-1 RNA in the genital tract (> 400 copies/mL by NucliSens). It was observed in ACTG 347/850 that 6% (one) of 17 men with undetectable blood plasma HIV-1 RNA (< 400 copies/mL by NucliSens) at week 16 had detectable seminal plasma viral load (HIV-1 RNA > 400 copies/mL by NucliSens). Dornadula et al. (5) reported that 45% (10) of 22 subjects (20 men and 2 women) with blood plasma HIV-1 RNA < 50 copies/mL had detectable viral HIV-1 RNA levels in genital fluids. Hence, power statements for the second primary objective are given for a variety of scenarios. As above, virologic failure is considered as a binary endpoint.

If 40% of these 100 subjects have detectable nonblood viral load, then there is 80% power to compare subsequent blood plasma virologic failure rates of 5% versus 26% (similarly 80% power for 10% versus 34% and for 15% versus 42%), comparing subjects with low versus high levels of a nonblood viral load measure at the time of suppressed blood viral load. If 20% of these 100 subjects have detectable nonblood viral load, then there is 80% power to compare subsequent blood plasma virologic failure rates of 5% versus 33% (similarly 80% power for 10% versus 41% and for 15% versus 49%).

If 10% of these 100 subjects have detectable nonblood viral load, then there is 80% power to compare subsequent blood plasma virologic failure rates of 5% versus 44% (similarly 80% power for 10% versus 53% and for 15% versus 62%). This is based on a two-tailed Fisher exact test, alpha = 0.05. Note that these power statements can be considered conservative, in that higher power is expected if the nonblood viral load can be modeled as a continuous variable (when it is detectable), or if time-to-event methods are used instead of the comparison of proportions (and more so if the virologic failure rate is higher).

7.33 Virus Detection

Prior data suggest that the probability of detecting virus from genital secretions differs between men and women with suppressed blood plasma HIV-1 RNA levels (with higher rates observed in women), so men and women will be assessed separately. It is assumed that 25 men and 25 women will remain on study through week 48, have a week 48 genital secretion specimen available, and have persistently suppressed blood plasma viral loads (< 200 copies/mL) from week 16 through week 48.

The following table describes 95% confidence intervals (in particular, showing their width) for various possible realizations of the data for the genital secretions aspect of the third primary objective.

Table 1. Confidence Intervals for a Percentage

| Observed | Percentage | Exact  95% CI |
| --- | --- | --- |
| 1/25 | 4% | 0 - 20% |
| 2/25 | 8% | 1 – 26% |
| 3/25 | 12% | 3 - 31% |
| 4/25 | 16% | 5 – 36% |
| 5/25 | 20% | 7 – 41% |
| 6/25 | 24% | 9 – 45% |
| 8/25 | 32% | 15 – 53% |
| 10/25 | 40% | 21 – 61% |
| 12/25 | 48% | 28 – 69% |
| 15/25 | 60% | 39 - 79% |

The above table may also be appropriate for assessing confidence interval width for detecting virus in lymph tissues among subjects with persistently suppressed blood plasma HIV-1 RNA levels (grouping men and women together, using the same assumptions as above and assuming that lymph tissue at week 48 will be available with one-half the probability of genital secretion specimens, thus yielding a total of 25 subjects).

Table 2 describes 95% confidence intervals for the saliva aspect of the third primary objective (grouping men and women together and using the same assumptions as for genital secretions, thus yielding a total of 50 subjects).

Table 2. Confidence Intervals for a Percentage

| Observed | Percentage | Exact  95% CI |
| --- | --- | --- |
| 2/50 | 4% | 0 – 14% |
| 4/50 | 8% | 2 – 19% |
| 6/50 | 12% | 5 – 24% |
| 8/50 | 16% | 7 – 29% |
| 10/50 | 20% | 10 – 34% |
| 12/50 | 24% | 13 – 38% |
| 16/50 | 32% | 20 – 47% |
| 20/50 | 40% | 26 – 55% |
| 24/50 | 48% | 34 – 63% |
| 30/50 | 60% | 45 - 74% |

## Monitoring Plan

Assessment of the availability (collection and proper shipment/storage) of the compartmental samples (and of blood viral loads) will be conducted periodically. Also, study accrual and follow-up will be assessed (overall, by gender, and by antiretroviral-naïve versus antiretroviral-experienced subjects).

## Analysis Plan

Because the men and women in this study may have different viral load levels, especially in genital secretions, gender-specific cutpoints may be used in the analysis. Sample size permitting, the influence of subjects’ baseline antiretroviral-naïve (< 1 week) versus antiretroviral-experienced status will be examined. In addition to the time-to-event analyses described below, interval-censored analysis methods will be used.

Baseline blood plasma viral load will be the average of the preentry and entry log_10_ HIV-1 RNA values.

7.51 Virologic Failure

The significance of baseline nonblood viral load in predicting subsequent virologic failure will be evaluated with Kaplan-Meier curves and in a Cox regression model that already includes a term for baseline blood viral load. A supplemental analysis will be performed in which subjects without blood viral load measures at week 16 or later will be considered censored in the analysis.

Subjects analyzed will be those with both a baseline blood plasma viral load and a baseline nonblood viral load.

7.52 Conditional Virologic Failure

The significance of nonblood viral load at the time of blood plasma viral suppression in predicting subsequent blood plasma virologic failure will be evaluated with Kaplan-Meier curves and the log-rank test. Additional analyses using the Cox regression model may also be conducted.

Subjects analyzed will be those with a week 16 nonblood viral load, with blood plasma HIV-1 RNA < 200 copies/mL at week 16 and a subsequent blood plasma viral load measure.

7.53 Virus Detection

Percentages will be described using exact confidence intervals. This will be done separately for men and women with respect to detecting virus in genital secretions.

Subjects analyzed will be those who have a week 48 specimen available and have persistently suppressed blood plasma viral loads (< 200 copies/mL) from week 16 through week 48.

7.54 Virologic Events

The assessment of differences in virologic events between blood and nonblood compartments will be based on the nonparametric sign test. Only those subjects in whom it can be determined which event (blood versus nonblood) occurred earlier will contribute to the analysis; the interval-censored nature of the data will be considered in making this determination.

# HUMAN SUBJECTS

## Institutional Review Board (IRB) Review and Informed Consent

This protocol and the informed consent document (Appendix VI) and any subsequent modifications will be reviewed and approved by the IRB or ethics committee responsible for oversight of the study. Written and signed informed consent will be obtained from the subject (or parent, legal guardian, or person with power of attorney for subjects who cannot consent for themselves, such as those below the legal age). The subject’s assent must also be obtained if he or she is able to understand the nature, significance, and risks associated with the study. The consent form will describe the purpose of the study, the procedures to be followed, and the risks and benefits of participation. A copy of the consent form will be given to the subject (or parent or legal guardian).

## Subject Confidentiality

All laboratory specimens, evaluation forms, reports, and other records will be identified by a coded number only to maintain subject confidentiality. All records will be kept in a locked file cabinet. All computer entry and networking programs will be done with coded numbers only. Clinical information will not be released without written permission of the subject, except as necessary for monitoring by the NIAID.

## Study Discontinuation

The study may be discontinued at any time by the NIAID.

# PUBLICATION OF RESEARCH FINDINGS

Publication of the results of this trial will be governed by ACTG policies.

# BIOHAZARD CONTAINMENT

As the transmission of HIV and other blood-borne pathogens can occur through contact with contaminated needles, blood, and blood products, appropriate blood and secretion precautions will be employed by all personnel in the drawing of blood and shipping and handling of all specimens for this study, as currently recommended by the Centers for Disease Control and Prevention.

All infectious specimens will be sent using the ISS-1 SAF-T-PAK mandated by the International Air Transport Association Dangerous Goods Regulations-Packing Instruction 602. Please refer to individual carrier guidelines, e.g., FedEx, Airborne, for specific instructions.

# REFERENCES

1. Department of Health and Human Services, Henry J. Kaiser Family Foundation. Guidelines for the use of antiretroviral agents in HIV‑infected adults and adolescents. http://www.hivatis.org 1999.

2. Wong JK, Hezareh M, Gunthard HF, et al. Recovery of replication‑competent HIV despite prolonged suppression of plasma viremia. Science 1997; 278:1291‑1295.

3. Finzi D, Hermankova M, Pierson T, et al. Identification of a reservoir for HIV‑1 in patients on highly active antiretroviral therapy. Science 1997; 278:1295‑1300.

4. Zhang L, Ramratnam B, Tenner‑Racz C, et al. Quantifying residual HIV‑1 replication in patients receiving combination antiretroviral therapy. N Engl J Med 1999; 340:1605‑1613.

5. Dornadula G, Zhang H. VanUitert B. et al. Residual HIV‑1 RNA in blood plasma of patients taking suppressive highly active antiretroviral therapy. JAMA 1999; 282:1627‑1632.

6. Furtado MR, Callaway DS, Phair JP, et al. Persistence of HIV‑1 transcription in peripheral‑blood mononuclear cells in patients receiving potent antiretroviral therapy. N Engl J Med 1999; 340:1614‑1622.

7. Embretson J, Zupancic M, Ribas JL, et al. Massive covert infection of helper T lymphocytes and macrophages by HIV during the incubation period of AIDS. Nature 1993; 362:359‑362.

8. Pantaleo G. Graziosi C, Demarest JF, et al. HIV infection is active and progressive in lymphoid tissue during the clinically latent stage of disease. Nature 1993; 362:355-358.

9. Haase AT, Henry K, Zupancic M, et al. Quantitative image analysis of HIV‑1 infection in lymphoid tissue. Science 1996; 274:985‑989.

10. Cavert W. Notermans DW, Staskus K, et al. Kinetics of response in lymphoid tissues to antiretroviral therapy of HIV‑1 infection. Science 1997; 276:960‑964.

1. Notermans DW, Jurriaans S. de Wolf F. et al. Decrease of HIV‑1 RNA levels in lymphoid tissue and peripheral blood during treatment with ritonavir, lamivudine and zidovudine. AIDS 1998; 12:167‑173.

REFERENCES (Cont.)

1. Hirsch MS, Conway B. D'Aquila RT, et al. Antiretroviral drug resistance testing in adults with HIV infection. JAMA 1998; 279:1984‑1991.

13. Wong JK, Gunthard HF, Havlir DV, et al. Reduction of HIV‑1 in blood and lymph nodes following potent antiretroviral therapy and the virologic correlates of treatment failure. Proc Natl Acad Sci USA 1997; 94:12574‑12579.

14. Gunthard HF, Wong JK, Ignacio CC, et al. Human immunodeficiency virus replication and genotypic resistance in blood and lymph nodes after a year of potent antiretroviral therapy. J Virol 1998; 72:2422‑2428.

15. Vernazza PL, Gilliam BL, Dyer J, et al. Quantification of HIV in semen; correlation with antiviral treatment and immune status. AIDS 1997;11:987-993.

16. Coombs RW, Speck CE, Hughes JP, et al. Association between culturable HIV-1 in semen and HIV-1 RNA levels in semen and blood: evidence for compartmentalization of HIV-1 between semen and blood. J Infect Dis 1998;177:320-330.

17. Gupta P, Mellors J, Kingsley L, et al. High viral load in semen of HIV-1 infected men at all stages of disease and its reduction by therapy with protease and non-nucleoside RT inhibitors. J Virol 1997;71:6271-6275.

18. Xu C, Politch JA, Tucker L, Mayer KH, Seage GR, Anderson DJ. Factors associated with increased levels of HIV-1 DNA in semen. J Infect Dis 1997;176:941-947.

19. Goulston C, McFarland W, Katzenstein D. Human immunodeficiency viruses type 1 RNA in the female genital tract. J Infect Dis 1998;177:1100-1103.

20. Uvin SC, Caliendo AM. Cervicovaginal human immunodeficiency virus secretions and plasma viral load in human immunodeficiency virus-seropositive women. Obstet Gynecol 1997; 90:739-743.

21. Iversen AK, Larsens AR, Jensen T, et al. Distinct determinants of human immunodeficiency virus type 1 RNA and DNA loads in vaginal and cervical secretions. J Infect Dis 1998; 177:1214-1220.

22. Hart CE, Lennox JL, Pratt-Palmore M, et al.. Correlation of human immunodeficiency virus type 1 RNA levels in blood and the female genital tract. J Infect Dis 1999;179:871-882.

REFERENCES (Cont.)

23. Fiore JR, Lepera A, Di Stefano M, et al. Frequent cervicovaginal shedding of HIV-1 in asymptomatic, non-severely immunodeficient women. AIDS 1999; 13:626-627.

24. Reichelderfer P, Coombs R, Wright D, Burns D, Kovacs A for WHS 001 Study Group, NIH. Impact of repeated sampling on detection of HIV-1 in the female genital tract. [Abstract I-251, p 120]. In: Abstracts of the 38th Annual ICAAC, San Diego, CA, September 24 ­ 27, 1998.

25. Coombs R, Wright D, Kovacs A, Burns D, Reichelderfer P. Sno-Strip™ wicking compared with cytobrush and cervicovaginal lavage for the quantification of HIV-1 nucleic acid in the female genital tract. [Abstract 1579, p 124]. In: Abstracts of the 39th Annual ICAAC, San Francisco, CA, September 26-29, 1999.

26. Reichelderfer P, Coombs R, Wright D, Burns D, Cohn J, Kovacs A. Variation in genital tract shedding of HIV RNA with the menstrual cycle. [Abstract 223, p 110]. In: Abstracts of the 6^th^ Conference on Retroviruses and Opportunistic Infections, Chicago, IL, January 31­February 4, 1999.

27. Tachet A, Dulioust E, Salmon D, et al. Detection and quantification of HIV-1 in semen: identification of a subpopulation of men at high potential risk of viral sexual transmission. AIDS 1999;13:823-831.

28. Gilliam BL, Dyer JR, Fiscus SA, et al. Effects of reverse transcriptase inhibitor therapy on the HIV-1 viral burden in semen. JAIDS 1997;15:54-60.

1. Eron JJ, Smeaton LM, Fiscus SA, et al. HIV-1 levels in semen: the effects of protease inhibitor therapy on HIV-1 levels in semen. J Infect Dis 2000;181:1622-1628.

30. Wong J, Gunthard H, Fiscus S, et al. Residual HIV RNA and DNA in lymph node and HIV RNA in genital secretions and in CSF after two years of suppression of viremia in the Merck 035 cohort. [Abstract 6]. In: Abstracts of the 6^th^ Conference of Retroviruses and Opportunistic Infections, Chicago, IL, January 31 - February 4, 1999.

31. Vernazza PL, Troiani L, Flepp MJ, et al. Potent antiretroviral treatment of HIV-infection results in suppression of the seminal shedding of HIV. AIDS (In press).

REFERENCES (Cont.)

32. Zhang H, Dornadula G, Beumont M, et al. Human immunodeficiency virus type 1 in the semen of men receiving highly active antiretroviral therapy. N Engl J Med 1998; 339:1803-1809.

33. Eron JJ, Vernazza PL, Johnston DM, et al. Resistance of HIV-1 to antiretroviral agents in the blood and seminal plasma: implications for transmission. AIDS 1998;12:F181-9.

34. Wainberg MA, Beaulieu R, Tsoukas C, Thomas R. Detection of zidovudine-resistant variants of HIV-1 in genital fluids. AIDS 1993;7:433-44.

35. DiStefano M, Fiore R, Monno L, et al. Detection of multiple drug-resistance-associated pol mutations in cervicovaginal secretions from women largely treated with antiretroviral agents. AIDS 1999;13:992-994.

36. Erice A, Mayers DL, Strike DG, et al. Primary infection with zidovudine-resistant human immunodeficiency virus type 1. N Engl J Med 1993;328:1163-1165.

37. Conlon CP, Klenerman P, Edwards A, Larder BA, Phillips RE. Heterosexual transmission of human immunodeficiency virus type 1 variants associated with zidovudine resistance. J Infect Dis 1994;169:411-415.

38. Imrie A, Beveridge A, Genn W, et al. Transmission of human immunodeficiency virus type 1 resistant to nevirapine and zidovudine. J Infect Dis 1997;175:1502-6.

39. Yerly S, Kaiser L, Race E, Bru J, Clavel F, Perrin L. Transmission of antiretroviral-drug-resistant HIV-1 variants. Lancet 1999;354:729-33.

40. Hecht FM, Grant RM, Petropoulos CJ, et al. Sexual transmission of an HIV-1 variant resistant to multiple reverse-transcriptase and protease inhibitors. N Engl J Med 1998; 339:307-11.

41. Yeung SCH, Kazazi F, Randle CGM, et al. Patients infected with human immunodeficiency virus type 1 have low levels of virus in saliva even in the presence of periodontal disease. J Infect Dis 1993;167:803-809.

42. Phillips J, Qureshi N, Barr C, Henrard D. Low levels of cell-free virus detected at high frequency in saliva from HIV-1-infected individuals. AIDS 1994; 8:1011-2.

REFERENCES (Cont.)

43. Liuzzi G, Chirianni A, Clementi M, et al. Analysis of HIV-1 load in blood, semen and saliva: evidence for different viral compartments in a cross-sectional and longitudinal study. AIDS 1996;10:F51-56.

44. Shugars DC, Slade GD, Patton LL, Fiscus SA. Oral and systemic factors associated with increased levels of human immunodeficiency virus type 1 RNA in saliva. Oral Surgery Oral Med Oral Pathol Oral Radiol and Endodon 2000;89:432-440.

1. Kovacs A, Reichelderfer P, for DATRI 009 Study Team. HIV is readily detectable in the female genital tract of HIV+ women. [Abstract 711]. In: Abstracts of the 5th Conference on Retrovirus and Opportunistic Infections, Chicago, IL, February 1-5, 1998.

SCHEDULE OF EVENTS

|  |  |  |  | WEEK | | | | | | | | | | | |  |
| --- | --- | --- | --- | --- | --- | --- | --- | --- | --- | --- | --- | --- | --- | --- | --- | --- |
| Evaluations | Screening | Pre-Entry | Entry | 8 | 16 | 24 | 32 | 40 | 48 | 56 | 64 | 72 | 80 | 88 | 96 | Virologic Failure/  Premature Discontinuation Visit |
| Informed consent | X |  |  |  |  |  |  |  |  |  |  |  |  |  |  |  |
| Documentation of HIV-1 infection | X |  |  |  |  |  |  |  |  |  |  |  |  |  |  |  |
| Plasma collection or documentation of HIV-1 RNA  > 2000 copies/mL within 60 days of study entry | X |  |  |  |  |  |  |  |  |  |  |  |  |  |  |  |
| Clinical assessment^1^ | X |  |  | X | X | X | X | X | X | X | X | X | X | X | X | X |
| Targeted physical exam^2^ |  |  | X |  |  |  |  |  |  |  |  |  |  |  |  |  |
| Medication history & updates^3^ | X |  | X | X | X | X | X | X | X | X | X | X | X | X | X | X |
| Pregnancy test (urine or serum ß-HCG)^4^ | X | Anytime pregnancy is suspected | | | | | | | | | | | | | |  |
| Hematology ^5^ |  | X | X | X | X | X | X | X | X | X | X | X | X | X | X | X |
| CD4/CD8 cell count^6^ |  | X | X | X | X | X | X | X | X | X | X | X | X | X | X | X |
| HIV-1 RNA (real time) ^6^ |  | X | X | X | X | X | X | X | X | X | X | X | X | X | X | X |
| Plasma/PBMC collection & storage^7^ |  |  | X |  | X |  |  |  | X |  |  |  |  |  | X | X |
| Saliva collection^8^ |  |  | X |  | X |  |  |  | X |  |  |  |  |  | X | X |
| Genital secretions collection^9^ |  |  | X |  | X |  |  |  | X |  |  |  |  |  | X | X |
| Lymphoid tissue collection^9^ |  |  | X |  | X |  |  |  | X |  |  |  |  |  | X | X |

^1^ Screening: complete clinical assessment to include a history of all HIV-related diagnoses and AIDS-defining events, and a signs and symptoms assessment with symptom-directed physical exam. If any signs or symptoms of a genitourinary tract infection are noted, the subject should be evaluated and treated for at least 14 days and the infection considered resolved prior to entry.

On Study/Virologic Failure/Premature Discontinuation Visits: clinical assessment to include updated signs/symptoms and new HIV-related diagnoses.

^2^ Targeted physical exam to include signs/symptoms and new HIV-related diagnoses. If any signs or symptoms of a genitourinary tract infection are noted, the subject should be evaluated and entry delayed until the subject has been treated for at least 14 days and the infection is considered resolved.

^3^ Medication history to include all anti-HIV medications, immune-based therapies, and HIV vaccines ever taken prior to study entry. Medication updates to include changes in anti-HIV medications, immune-based therapies, and HIV vaccines.

^4^ Pregnancy test must be performed within 14 days of entry and any time pregnancy is suspected.

^5^ CBC with differential required only if CD4/CD8 cell counts will be measured at this visit.

^6^ If CD4/CD8 cell counts or HIV-1 RNA levels are measured as part of a coenrolled study at the same time as an A5077 protocol visit, or total blood volumes exceed safe limits, they do not need to be repeated for A5077. If available, the results should be obtained and entered onto the appropriate A5077 CRF. If HIV-1 RNA levels are measured as part of A5077, see Appendix II for collection, processing, storage, and shipment information.

^7^ Required unless total blood volumes exceed safe limits. See Appendix II for collection, processing, and storage information.

^8^ Saliva collection required of all subjects (see Appendix III).

^9^ Subjects have the option to contribute either genital secretions or lymphoid tissues, or both (see Appendix IV and V).

BLOOD COLLECTION, PROCESSING, STORAGE, AND SHIPMENT PROCEDURES

1.0. OVERVIEW OF VIROLOGIC EVALUATIONS

Unless total blood volumes exceed safe limits (the American Red Cross has a limit of 450 mL of blood over 56 days for healthy volunteers), all subjects will have blood samples obtained for real-time HIV-1 RNA levels at preentry, entry, week 8 and every 8 weeks thereafter until week 96, within 30 days of confirmed virologic failure, and at the premature study discontinuation visit. Plasma and PBMCs will be collected and stored at entry, weeks 16, 48, and 96, within 30 days of confirmed virologic failure, and at the premature study discontinuation visit.

NOTE: If total blood volumes exceed safe limits and do not permit an A5077 blood draw, the A5077 protocol team will prioritize requests for access to specimens in the following order of priority: 1) one plasma aliquot (1.5 mL); 2) one aliquot of frozen PBMCs (> 2 x 10^6^ cells).

Virologic studies planned include the following:

- Quantitation of HIV-1 RNA levels in plasma using the UltraSensitive Roche assay.
- Genotypic resistance studies (analysis of HIV-1 reverse transcriptase and protease sequences) in plasma.
  NOTE: DNA extracted from PBMC will be used for genotypic resistance studies when plasma HIV-1 RNA levels are low (< 1000 HIV RNA copies/mL).
- Detection and quantitation of HIV-1 proviral DNA (total and 2-LTR circles) in PBMC fractions using an in-house assay.

2.0 SPECIMEN COLLECTION, PROCESSING, AND STORAGE

NOTE: SPECIMENS MUST BE DELIVERED TO THE LABORATORY AND PROCESSED WITHIN 48 HOURS OF COLLECTION

A. Specimen Collection

ONE 10.0 mL purple-top EDTA tube (Becton Dickinson re-order number #6457) will be drawn from all subjects at preentry, entry, week 8 and every 8 weeks thereafter until week 96, within 30 days of confirmed virologic failure, and at the premature study discontinuation visit.

B. Specimen Processing

1. Clarified plasma

To minimize RNA degradation, all plasma samples must be processed and stored at -70°C within 48 hours of collection. Although the required processing time has been increased from 4-6 hours to 48 hours, sites are encouraged to continue to process HIV-1 RNA samples as soon as possible. Specifically, the EDTA tube should be centrifuged at low speed (400 x *g*) for 10 minutes at room temperature as soon as possible after collection.

Plasma should be carefully removed from each tube, placed in a sterile polypropylene conical centrifuge tube and centrifuged again (at 800 x *g*) for 10 minutes to completely remove platelets and cell debris. Store as many 1.2 mL aliquots of plasma as possible at –70°C.

2. PBMCs

After plasma has been removed as indicated above, PBMCs will be obtained and stored according to procedures outlined in the AACTG Virology Manual for HIV Laboratories (January 1997 version).

Store TWO TO FOUR aliquots of 2 x 10^6^ cells as NON-VIABLE DRY cell pellets at - 70°C.

C. Real Time Plasma HIV-1 RNA

NOTE: Participating sites should try to avoid virologic sampling for confirmatory testing during an intercurrent illness, within 14 days after a subject receives an immunization or within 14 days of a dose interruption, as this may result in a transient elevation of plasma HIV-1 RNA levels.

Within ten (10) business days following the receipt of a plasma sample(s) by the ACTG-specified laboratory for the performance of real time plasma HIV-1 RNA assays, the designated laboratory will FAX the results to the clinic. Upon receipt, the clinic is to key the results into the central ACTG database.

If the result provides the first suggestion of a virologic failure, the subject should be recalled to the clinic to have a confirmatory virologic (plasma) specimen obtained. Every attempt should be made to have the confirmatory sample drawn as soon as possible, but no later than 30 days after the date of the first sample.

Results confirming study endpoints should be referred for verification as soon as possible to the protocol team via the actg.teama5077 e-mail group, to the attention of the protocol chair or vice chair(s). When sending a request for verification of a study endpoint, the site should include the following information: PID, HIV-1 RNA values, and date of draws for 1) the first time point of failure and 2) the confirmatory sample.

TWO 1.2 mL aliquots of plasma for HIV-1 RNA determinations must be shipped (frozen on dry ice) via overnight, priority express to the contracting laboratory at Johns Hopkins University (JHU). Shipping instructions and how to obtain shipping materials are provided below.

All aliquot vials should be labeled with the PID, study number, unique identifier, VID, date drawn, and derivative (p12)/anticoagulant (EDTA).

All plasma HIV-1 RNA assays will be done at Dr. Brooks Jackson's laboratory at JHU using the Roche UltraSensitive assay. Dr. Jackson's laboratory will send sites the proper shipping containers and supplies. Sites need to contact the JHU laboratory (phone number below) to ask that shipping materials be sent. When calling, please provide the contact person and shipping address where the materials are to be sent.

For shipments of more than 15 vials, the 1.2 mL plasma samples should be placed in the Nalgene® 5x5 or 9x9 cryovial box in the order of the shipping manifest (if the LDMS program is available). If fewer than 15 vials are to be sent, they can be placed in plastic baggies inside the orange-top shipping container. Each vial should be individually bagged or wrapped prior to placing in baggies. The 5x5 cryovial box should be securely covered and placed in the orange-top container or in an IS2 certified (STP 370) shipping container filled with carbon dioxide (dry ice).

The orange-top container should have absorbent material included. The container is then placed in the cardboard box, which is placed inside the polystyrene insulated box and the box filled with sufficient dry ice to last at least 48 hours. DO NOT PLACE DRY ICE INSIDE THE ORANGE-TOP CANISTER! Likewise, the Nalgene® 9x9 or 5x5 cryovial box should be wrapped in absorbent material and placed inside a shipping plastic bag, sealed, and placed in an IS2 certified (STP 370) shipping container filled with dry ice.

The outer box is sealed with tape and marked with the appropriate stickers. Follow IATA/ICAO and ACTG shipping guidelines for the shipment of infectious (see section 6.2) materials. The Virology Tracking Form should accompany the specimens to the laboratory.

D. Paperwork

An LDMS shipping diskette, shipping manifest, and box map (if appropriate) must accompany the shipment as well as the tracking form. This material should not be placed in the dry ice, but in a plastic bag between the polystyrene container and outer box or outside the foam area of the IS2 certified shipping container. If the site does not have the LDMS, an ACTG Virology shipment notice, duplicate Virology Tracking Form, and a list of specimens being sent (box map) should be included with the shipment. ALWAYS include an inventory with every shipment. The inventory must include complete information--PID, study number, draw date, VID (if week 0, indicate if screen, preentry, or entry), clinic number, number of aliquots, volume, position in box, AND sending laboratory information. The inventory may be in the form of a spreadsheet, typed or legibly handwritten. (Mini-programs are available through FSTRF to create both spreadsheets and diskettes.)

The samples and appropriate paperwork should be sent to:

Dr. Brooks Jackson

ATTN: Estelle Piwowar-Manning

Department of Pathology - Johns Hopkins University

Room 313 Pathology Building

600 North Wolfe Street

Baltimore, MD 21287

Phone: 410-502-5296

FAX: 410-614-0430

E-mail: epiwowa@jhmi.edu

Prior to shipment, please FAX the ACTG Virology Shipping Notice to Estelle Piwowar-Manning at 410-614-0430. If the FAX does not seem to be transmitting, please call the laboratory at 410-502-5296 to advise that a shipment has been sent and provide the airway bill number. Any questions relating to specimen handling, shipping, or identification should be directed to Estelle Piwowar-Manning at 410-614-6736.

Please call the laboratory around holiday times to check available days for shipping.

Shipments may be made Monday through Wednesday only by overnight courier service (Federal Express) and billed to the recipient (Fed Ex acct. number 2182-1427-4). The laboratory is not open to receive shipments on weekends or holidays. The Federal Express forms will be provided.

E. Specimen Storage

All other plasma and PBMC aliquots are to be stored frozen and shipped to the repository per the AACTG guidelines (see F below).

F. Instructions for Shipping to the Repository

Unless otherwise stated, stored samples should be shipped to the repository. Please refer to the AACTG web site at: <http://aactg.s-3.com/specrepos.htm> for complete instructions on packaging, shipping, and repository notification.

SALIVA COLLECTION, PROCESSING, STORAGE, AND SHIPMENT PROCEDURES

1.0 MATERIAL NEEDED

- 50-mL sterile centrifuge tube with screw-on cap
- Plastic or paper cup containing wet ice
- 2.0-mL cryostorage tubes with screw-on caps
- Labels
- Sterile pipettes
- Aerosol-resistant pipette tips
- Slow-speed table-top centrifuge

2.0 SPECIMEN COLLECTION

All study subjects will have saliva samples collected at study entry, weeks 16, 48, and 96, within 30 days of confirmed virologic failure, and at the premature study discontinuation visit.

After obtaining informed consent, ask the subject to expectorate into a sterile 50-mL centrifuge tube, which is kept cool on wet ice. Note that the subject is not to have eaten or brushed his/her teeth within 30 minutes of sample collection. A total of 5 mL of whole unstimulated saliva should be collected (procedure may take 5-15 minutes depending on subject’s salivary flow rate). Label the tube with PID, SID, and date. The sample should be frozen at -20ºC within 2 hours of collection and stored on site at -80° C until shipped on dry ice to the repository (see section 4.0).

3.0 SPECIMEN PROCESSING

1. Measure the sample volume and record (in mLs).

2. Vortex the specimen briefly and then transfer two 1-mL aliquots of whole saliva to sterile labeled cryostorage tubes using a sterile 5-mL pipette. This untreated saliva is referred to as “whole saliva.”

3. Salivary cells are recovered from the remaining saliva sample as follows. Recap the tube containing the sample and centrifuge at 400 x *g* at 4°C for 10 minutes to pellet the cells, cellular debris, and mucins.

4. Remove the supernatant with a 5-mL pipette and transfer 1-mL aliquots to sterile, labeled cryovials. Label as cell-free saliva.

5. Add 30 mL cold sterile phosphate-buffered saline (PBS) to the tube containing the saliva cell pellets, recap the tube, and resuspend the pellets by gently vortexing for 10-15 seconds.

6. Centrifuge the cell suspension at 400 x *g* at 4°C for 5 minutes.

7. Remove and discard the supernatant.

8. Repeat the washing procedure once more.

9. After the final wash, remove all of the supernatant. Resuspend the cell pellets in 1 mL of cold PBS, remove a small aliquot (~20 µL), and count the cells using the hemocytometer and record.

10. Transfer the cell suspension to sterile cryovials in 0.5-mL aliquots.

4.0 SPECIMEN STORAGE AND SHIPMENT

Samples should be stored on-site at -80°C and shipped to the repository. Please refer to the AACTG web site at: <http://aactg.s-3.com/specrepos.htm> for complete instructions on packaging, shipping, and repository notification.

5.0 ANALYSIS

Virologic studies planned include the following:

- Quantitation of HIV-1 RNA levels in saliva using the Organon Teknika NucliSens™ HIV-1 RNA QT assay.
- Detection and quantitation of HIV-1 proviral DNA (total and 2-LTR circles) in salivary cells using an in-house assay.
- Genotypic resistance studies (analysis of HIV-1 reverse transcriptase and protease sequences) in saliva.
  NOTE: DNA extracted from salivary cells will be used for genotypic resistance studies when HIV-1 RNA levels in saliva are low (< 1000 HIV RNA copies/mL).

GENITAL SECRETIONS COLLECTION, PROCESSING,

STORAGE, AND SHIPMENT PROCEDURES

Subjects have the option to contribute either genital secretions or lymph tissues, or both, at study entry, weeks 16, 48, and 96, within 30 days of confirmed virologic failure, and at the premature study discontinuation visit.

1.0 FEMALE SUBJECTS

1.1 Specimen Collection

The subject must refrain from any kind of sexual activity, douching, and inserting any intravaginal products for at least 48 hours prior to the collection of vaginal/cervical specimens. Samples will be collected by the following methods in the following order: Sno-Strip™, cervicovaginal lavage (CVL), cytobrush, and only for subjects coenrolled in A5029, a Pap smear will also be performed.

1.11 Endocervical canal fluid collected by Sno-Strip wicking. The purpose of this collection procedure is to obtain endocervical canal fluid for viral RNA quantification.

1. Gently insert an unlubricated speculum into the vagina.

2. Sno-Strips will be used as wicks to collect primarily cell-free virions from the endocervical fluid. If excess mucus or menses clot has accumulated near the cervical os, a large cotton-tipped cotton swab may be used to gently remove this material before inserting the Sno-Strips™.

3. Using forceps (ring or sponge forceps work well), gently insert three Sno-Strips simultaneously into the vagina, place through the cervical os into the distal endocervical canal, and hold in place to adsorb sample. Each Sno-Strip adsorbs approximately 8-µL of specimen. Adsorption usually takes approximately one minute, but may take a little longer.

4. Hold the narrow end of the three strips over and slightly inside one labeled plastic transport tube (1.5-mL cryovial) containing 500 µL of NASBA lysis buffer (provided by the University of North Carolina Retrovirology Laboratory). Cut the strips at the junction of the shoulder and neck of the Sno-Strip with scissors, allowing the narrow end to fall into the cryovial tube buffer.

5. Send the sealed vial to the local laboratory for processing.

1.12 Ectocervicovaginal lavage. The purpose of this collection procedure is to obtain a washing of virus and cells from the ectocervix and fluid from the posterior vaginal fornix for viral and immunologic studies.

1. Collect the ectocervicovaginal lavage by placing 10 mL of either normal saline or phosphate buffered saline in a 10-mL Luer-Lok® syringe.

2. Cut a sterile plastic transfer pipette just below the bulb, throw the bulb away and place the pipette tip on the syringe. Alternatively, a 14-gauge angiocath can be inserted over the tip of a 10-mL syringe. It may be helpful to seal the junction with parafilm.

3. Introduce the syringe through the speculum at the opening of the cervical os, but do not insert into os.

4. Aim a continuous stream of saline directly at and into the os to bathe the cervix and the ectocervix.

5. Allow the fluid to pool into the posterior fornix and aspirate into the same syringe.

6. Repeat this procedure exactly 5 times with the same fluid; do not add any additional saline or PBS to the specimen.

7. Transfer the fluid to a sterile 15-mL conical test tube.

8. Transport to the virology laboratory within 1 hour of collection. If this is not possible, place specimens on ice and refrigerate until transport up to 4 hours.

NOTE: For subjects coenrolled in A5029, the lavage fluid will be divided in half prior to centrifugation. Half will be processed as above, and half will be processed as specified in A5029.

1.13 Endocervical canal cytobrush. The purpose of this collection procedure is to obtain primarily cells for viral DNA quantification.

NOTE : For subjects coenrolled in A5029, a total of three cytobrush specimens will need to be collected. One should be collected and processed according to the instructions below for A5077 and the other two should be collected and processed according to the instructions in A5029.

1. Gently insert a cytobrush with a plastic shaft 1cm into the cervical os and rotate exactly 360 degrees. Bleeding usually occurs with the cytobrush and this should be noted.

2. Cytobrushes must be placed in the appropriate vials before drying occurs. The end of the cytobrush is snapped off with scissors so that the brush portion can fit into the transport tube. The cytobrushes snap easily, particularly if they are scored with a pair of scissors approximately 2 cm from the brush end of the handle. Place the brush in the vial so that the scored area is approximately even with the lip of the vial, hold the bottom of the handle and tube with one hand while snapping off the top of the handle with the other. Firmly tighten the lid of the cryovial, which already contains 0.5 mL of 4 M guanidinium isothiocyanate + mecaptoethanol. Cytobrushes in this fluid can remain at 4^o^C for up to 72 hours. Clinicians should send the sealed vial to the local laboratory for processing.

1.14 Pap Smears. This procedure should only be performed for subjects coenrolled in A5029. Please refer to the A5029 protocol for collection and processing instructions.

1.2 Specimen Processing

1. Sno-Strip and cytobrush specimens: At the processing laboratory, vortex each vial for 5 seconds and freeze upright at -70^o^C (it is not necessary to remove either the Sno-Strips or the cytobrush from the vials). At the testing laboratory, thaw and microfuge each vial at the highest microfuge speed for exactly 1 minute, remove and discard either the Sno-Strips or cytobrush, and quantitate the extracted nucleic acid.

2. Cervicovaginal lavage: In the laboratory note volume, color, and presence of gross blood or mucous in the specimens.

NOTE: For subjects coenrolled in A5029, the lavage fluid will be divided in half prior to centrifugation. Half will be processed as stated above in section 1.12, and half will be processed as specified in A5029.

3. Centrifuge the specimens at 600-800 x *g* for 10 minutes.

4. Aspirate the supernatant (including any mucous) and store as 2.0-mL aliquots, label and store at – 70^o^C.

5. Resuspend the cell pellet in 10 mL of PBS.

6. Centrifuge at 600 x *g* for 10 minutes.

7. Repeat steps 6 and 7 once and resuspend cells in 1 mL of PBS.

8. Divide one-half of the cell suspension (0.5 mL) between two labeled microfuge tubes; i.e., 0.25-mL/microfuge tube.

9. Centrifuge these cell suspensions at the highest speed in a microfuge for 3 minutes.

10. Aspirate the supernatants and store at -70^o^C as dry cell pellets.

11. Take the remaining 0.5-mL cell suspensions and cryopreserve using the Immunology Consensus Freezing Protocol. This specimen will be stored in liquid nitrogen.

1.3 Analysis

Virologic studies planned include the following:

- Quantitation of HIV-1 RNA levels in cervical fluid (Sno-Strip) using the Ultrasensitive Roche **assay**.
- Genotypic resistance studies (analysis of HIV-1 reverse transcriptase and protease sequences) in CVL. NOTE: DNA extracted from cervical cells will be used for genotypic resistance studies when HIV-1 RNA levels in CVL are low (< 1000 HIV RNA copies/mL).
- Detection and quantitation of HIV-1 proviral DNA (total and 2-LTR circles) in cervical cells (cytobrush) using an in-house assay.

2.0 MALE SUBJECTS

2.1 Specimen Collection

The subject must refrain from any kind of sexual activity for 48 hours prior to donation of semen. Subjects may collect semen specimens either at home or at the clinic. If a subject collects semen at home, he should be instructed to bring the specimen to the clinic within 3 of collection as processing must occur within 4 hours of collection. Ask the subject to follow the following collection procedure:

1. The glans (or head) of the penis should be cleaned with a Rantex™, or similar cleansing towel as used for a clean-catch urine sample, to remove dead cells and loose bacteria.

2. Masturbation into a sterile urine container--without the use of lubricants other than water--is required. Following masturbation, the semen specimen should sit for at least 30-45 minutes, BUT NO MORE THAN 4 HOURS, prior to processing to allow liquefaction of the semen.

2.2 Specimen Processing

1. Transfer the sample to a 15-mL conical centrifuge tube using a pipette, and measure and record the volume.

2. Centrifuge the semen at 600-800 x *g* for 10 minutes.

3. Remove the supernatant, aliquot into 0.5-mL aliquots in labeled cryovials and store at -70^o^C.

4. Resuspend the seminal cell pellets in 8 mL of PBS.

5. Layer onto 4 mL of Ficoll-Hypaque solution.

6. Centrifuge at 600 x *g* for 20 minutes.

7. Carefully remove nonspermatozoal mononuclear cells found at the interface and place in 10 mL of PBS.

8. Centrifuge at 600 x *g* for 10 minutes.

9. Resuspend in 10 mL of PBS.

10. Count cells and check viability using the trypan blue dye exclusion. An accurate cell count of the white blood cells will be difficult as they cannot be readily distinguished from immature germ cells. Record the number of nonspermatozoal mononuclear cells.

11. Centrifuge at 600 x *g* for 10 minutes.

12. Resuspend the seminal cell pellets in 1 mL of PBS.

13. Divide one-half of the cell suspension (0.5 mL) between two labeled microfuge tubes; i.e., 0.25-mL/microfuge tubes.

14. Centrifuge these cell suspensions at the highest speed in a microfuge for 3 minutes.

15. Aspirate the supernatants and store at -70^o^C as dry cell pellets.

16. Freeze the remaining cells using the Immunology Consensus Protocol for freezing viable cells.

17. Either freeze the cells using a step down freezer, or place in a Mr. Frosty or polystyrene test-tube rack for 2-24 hours at -70^o^C. Once frozen, transfer to liquid nitrogen storage.

2.3 Analysis

Virologic studies planned include the following:

- Quantitation of HIV-1 RNA levels in seminal plasma using the UltraSensitive Roche assay.
- Genotypic resistance studies (analysis of HIV-1 reverse transcriptase and protease sequences) in seminal plasma. NOTE: DNA extracted from seminal cells will be used for genotypic resistance studies when HIV-1 RNA levels in seminal plasma are low
  (< 1000 HIV RNA copies/mL).
- Detection and quantitation of HIV-1 proviral DNA (total and 2-LTR circles) in seminal cells using an in-house assay.

3.0 SHIPPING INSTRUCTIONS

All samples are to be stored frozen and shipped to the repository per the AACTG guidelines. Please refer to the AACTG web site at: <http://aactg.s-3.com/specrepos.htm> for complete instructions on packaging, shipping, and repository notification.

4.0 COMPENSATION

Although such a decision is ultimately left to local site discretion, it is highly recommended that compensation to subjects for collection of genital secretions be considered by all participating sites.

LYMPHOID TISSUE (LT) COLLECTION, PROCESSING, AND

SHIPPING PROCEDURES

Subjects have the option to contribute either genital secretions or lymph tissues, or both, at study entry, weeks 16, 48, and 96, within 30 days of confirmed virologic failure, and at the premature study discontinuation visit.

1.0 COLLECTION PROCEDURES

Because of the different methods that may be available at each site for sampling lymphoid tissue (LT) (e.g., tonsillar biopsy, lymph node biopsy, fine needle aspiration, endoscopic biopsy of gut-associated lymphoid tissue [GALT]), and the variety of assays used to detect, characterize, and quantitate HIV-1 RNA and DNA in LT specimens, it is important to study all LT specimens obtained from participants in AACTG trials using the same laboratory methods.

Current plans are to quantitate and characterize the HIV-1 RNA and HIV-1 DNA in all LT samples collected from the participants in this study. In addition, LT specimens will be studied by in situ hybridization at the University of Minnesota. Ideally, two fragments of LT should be obtained from all study participants at each time point: one fragment will be processed for HIV-1 RNA and DNA, and the remaining fragment will be processed for in situ hybridization studies. Please follow the specific processing and shipping instructions below.

The preferred procedures for obtaining LT samples are 1) lymph node biopsies and 2) tonsillar biopsies. Other methods (fine needle aspiration, endoscopic biopsy of GALT), might provide appropriate samples as well. Sites are encouraged to contact the protocol chair, Alejo Erice, M.D., to discuss sampling methods. Lymph tissue biopsies must be performed by a doctor at the site who has expertise or specializes in the procedure (e.g., an ENT for tonsillar biopsies).

Below are brief descriptions of suggested procedures for tonsillar biopsies and lymph node aspirates.

1.1 Tonsillar Biopsy Procedure

NOTE: When performing tonsillar biopsies, it is very important to sample from DEEP within the tonsil in order to obtain appropriate tissue fragments. Superficial biopsies contain mucosal epithelium for the most part and are NOT VALID for LT studies. Ideally, LT fragments of at least 25 mg should be obtained; larger (or multiple smaller) samples are preferred.

The ear, nose, and throat (ENT) consultant at each participating site should evaluate the subject.

1) The medical history should be reviewed to confirm that no aspirin, NSAIDs, or other medications have been taken within 2 weeks prior to the procedure, as these medications may affect coagulation.

2) The subject should be seated in an upright position in a standard examination chair with headrest for comfort and security. Topical anesthetic (10% xylocaine) should be sprayed over the tonsillar pillars, the tonsils, and soft palate.

3) This should be followed by infiltration of 0.5 cc of 2% xylocaine containing epinephrine (1:80,000 dilution) using a 30-gauge needle into the peritonsillar space behind the superior pole of the tonsil. After three minutes, anesthesia and gag reflex should be tested by stimulation of the tonsil area with a tongue blade. A second administration of anesthetic may be needed to achieve complete anesthesia, although this is rare.

4) A tonsillar tissue sample should be obtained from the superior pole of the tonsil using triangular adenoid punch biopsy forceps. Tonsillar tissue that is obtained during each biopsy procedure should be approximately 1 cc or 1/8 inch in size and divided into equal portions as previously indicated.

5) A chemical cautery (AgNO_3_) applicator followed by cold water oral rinses should be used to achieve hemostasis at the biopsy site.

6) Each subject should be monitored in the clinic for an additional 30-60 minutes in order to assess any excess pain or bleeding from the tonsils. If needed, subjects may be offered prescription or nonprescription pain medication or anesthetic spray.

1.2 Lymph Node Aspiration Procedure and Processing

Sites are encouraged to use persons with expertise in this procedure at their institution to obtain the best samples. Below is a suggested procedure:

1) The medical history should be reviewed to confirm that no aspirin, NSAIDs, or other medications have been taken within 2 weeks prior to the procedure, as these medications may affect coagulation.

2) Cleanse and anesthetize skin.

3) Pre-fill a 3-mL syringe with 0.5 mL sterile PBS-A (PBS-A is PBS without CA++).

4) Use a separate 5-mL syringe and a 21-gauge needle to perform the aspirate.

5) Replace the 5-mL syringe with a PBS-filled syringe and gently expel the sample/PBS-A into a 1.5-mL cryo tube.

6) Snap freeze the samples in liquid nitrogen and store at -70°C.

2.0 LYMPHOID TISSUE PROCESSING INSTRUCTIONS

LT fragments should be processed IMMEDIATELY after extraction from participants.

If two separate fragments have NOT been obtained, all LT specimens of sufficient size (EXCLUDING LYMPH NODE ASPIRATES, SEE SECTION 1.2 ABOVE) MUST be divided into at LEAST TWO fragments of equal proportion and processed according to the instructions below.

If only one small LT sample (less to or equal to 25 mg in weight) has been obtained, the entire fragment should be processed only for HIV-1 RNA and HIV-1 DNA studies, as outlined in section 2.1.

2.1 Processing of LT Samples for HIV-1 RNA and HIV-1 DNA Studies

One LT fragment will be used to make preparations for RNA and DNA quantitative and genotypic studies. These fragments should be processed as follows:

• Snap-freeze the LT fragment in liquid nitrogen and store WITHOUT PRESERVATIVES at -70°C until shipped.

• If preferable for the site, LT specimens can be shipped in batch every 12 weeks.

2.2 Processing of LT Samples for in situ Hybridization Studies

2.21 Small LT Samples (these include samples other than lymph node biopsies, such as tonsillar biopsies)

• Place the LT fragment into a sterile microcentrifuge containing Streck’s Tissue Fixative. The LT specimen should be completely immersed in the fixative. Fix specimen at room temperature for 24 hours.

NOTE: Streck’s Tissue Fixative can be purchased from Streck Laboratories, Inc. Phone: 800-843-0912, Catalog # 265138.

• Transfer the LT fragment to a fresh sterile microcentrifuge tube containing 80% ethanol. Store at room temperature. LT samples can be kept in 80% ethanol for up to 7 days.

• Paraffin-embed the LT fragment using existing standard methodologies.

NOTE: LT specimens can be shipped in 80% ethanol at room temperature or as paraffin-embedded blocks. Specimens in 80% ethanol, MUST be shipped within 7 days of collection.

2.22 Large LT Samples (e.g., lymph node biopsies)

• Place LT fragment into a sterile container containing 4% paraformaldehyde. The LT specimen should be completely immersed in the fixative. Fix specimen at room temperature for 24 hours.

NOTE: The 4% paraformaldehyde should be prepared fresh as follows:

• After weighing the paraformaldehyde, add half of the calculated volume of PBS required to prepare a 4% solution of paraformaldehyde in PBS (v/w).

• To help dissolve the paraformaldehyde, alkalinize the solution with concentrated NaOH (normally it would be necessary to reach a pH of 13).

• Adjust to a pH of 7.10 to 7.20 with concentrated HCl.

• Add PBS to bring up the volume to reach a final paraformaldehyde concentration of 4% (v/w).

• Drip-filter the solution through a #1 filter.

• After fixation in paraformaldehyde, transfer the LT fragment to a sterile container containing 80% ethanol. Store at room temperature. LT samples can be kept in 80% ethanol for up to 7 days.

• Paraffin-embed the LT fragment using existing standard methodologies.

NOTE: LT specimens can be shipped in 80% ethanol at room temperature or as paraffin-embedded blocks. Specimens in 80% ethanol, MUST be shipped within 7 days of collection.

3.0 ANALYSES PLANNED

- Quantitation of HIV-1 RNA levels in lymphoid tissue RNA extracts using the UltraSensitive Roche Amplicor HIV-1 Monitor™ assay.
- Genotypic resistance studies (analysis of HIV-1 reverse transcriptase and protease sequences) in lymphoid tissue RNA extracts.
- Quantitation of HIV-1 RNA and HIV-1 DNA in lymphoid tissue sections for in situ hybridization and quantitative image analysis using an in-house assay.

4.0 SHIPPING INSTRUCTIONS

LT specimens should be shipped to:

University of Minnesota HIV Laboratory

15-119PWB

516 Delaware Street, S.E.

Minneapolis, MN 55455

Laboratory Contacts:

Dr. Alejo Erice Max Schmeling

TEL: 612-626-0920 TEL: 612-626-5748

FAX: 612-625-5468 FAX: 612-625-5468

E-MAIL: [erice001@tc.umn.edu](mailto:erice001@tc.umn.edu) E-MAIL: [schme002@gold.tc.umn.edu](mailto:schme002@gold.tc.umn.edu)

SAMPLE INFORMED CONSENT

ADULT ACTG TRIALS

DIVISION OF AIDS, NIAID, NIH

ACTG A5077

REMINDER TO CLINICAL SITES: DO NOT USE PREAMBLE IN LOCAL CONSENTS

NOTE FROM OHRP (OFFICE FOR HUMAN RESEARCH PROTECTIONS) TO SITES ENROLLING SUBJECTS IN THIS STUDY:

---------------------------------------------------------------------------------------------------------------------

PLEASE NOTE THAT THIS SAMPLE LANGUAGE DOES NOT PREEMPT OR REPLACE LOCAL IRB REVIEW AND APPROVAL. INVESTIGATORS ARE REQUIRED TO PROVIDE THE LOCAL IRB WITH A COPY OF THIS SAMPLE LANGUAGE ALONG WITH THE LANGUAGE INTENDED FOR LOCAL USE. LOCAL IRBS ARE REQUIRED TO WEIGH THE UNIQUE RISKS, CONSTRAINTS, AND POPULATION CONSIDERATIONS AS A CONDITION OF ANY APPROVAL. ANY DELETION OR SUBSTANTIVE CHANGE OF INFORMATION CONCERNING RISKS OR ALTERNATIVE TREATMENT MUST BE JUSTIFIED BY THE INVESTIGATOR, APPROVED BY THE LOCAL IRB, AND NOTED IN THE IRB MINUTES. JUSTIFICATION AND IRB APPROVAL OF SUCH CHANGES MUST BE FORWARDED TO THE SITE REGISTRATION OFFICE FOR ANY DAIDS-SPONSORED TRIAL OR ANY OTHER NIH-SPONSORED TRIAL AS MAY BE OTHERWISE SPECIFIED. SPONSOR-APPROVED CHANGES IN A DAIDS PROTOCOL MUST BE APPROVED BY THE LOCAL IRB BEFORE USE UNLESS INTENDED FOR THE ELIMINATION OF APPARENT IMMEDIATE HAZARD. NEW INFORMATION SHALL BE SHARED WITH EXISTING SUBJECTS AT NEXT ENCOUNTER, WITH ALL NEW SUBJECTS PRIOR TO INVOLVEMENT, OR AS THE LOCAL IRB MAY OTHERWISE ADDITIONALLY REQUIRE.

---------------------------------------------------------------------------------------------------------------------

TITLE OF CLINICAL TRIAL: ACTG A5077, “VIROLOGIC STUDIES IN COMPARTMENTAL SAMPLES FROM HIV-INFECTED SUBJECTS CHANGING OR INITIATING POTENT ANTIRETROVIRAL THERAPY,” FINAL Version 1.0, dated 11/02/00.

PRINCIPAL INVESTIGATOR: PHONE:

INFORMED CONSENT

You are being asked to take part in the research study named above because you are infected with HIV, the virus that causes AIDS. This study will look at the type and amount of HIV in certain places in the body (called compartments) and in the blood of subjects who are changing or starting potent antiretroviral therapy. Before you decide whether or not to take part in this study, we would like to explain the purpose of the study, any risks to you, and what is expected of you.

YOUR PARTICIPATION IS VOLUNTARY

This consent form gives you information about the study which will be discussed with you. Once you understand the study, and if you agree to take part, you will be asked to sign this consent form. You will be given a copy of it to keep.

Before you learn about the study, it is important that you know the following:

• Your participation is entirely voluntary;

• You may decide not to take part in or to withdraw from the study at any time without losing the benefits of your routine medical care.

PURPOSE OF STUDY

The purpose of this study is to gain information on how the type and amount of HIV present in certain places in the body and in the blood are affected when potent (powerful) anti-HIV drugs are taken. We do not know the importance of the type or amount of HIV found in different areas of your body and its effect on your HIV disease.

You will be asked to give samples of blood, saliva, and either genital secretions or lymphoid tissues, or both, for this study.

About 164 people will take part in this study. You will be in this study for about 2 years.

PROCEDURES

Eligibility/Screening

If you decide to take part in this study and sign this consent form, you will be asked questions about how you are feeling, your medical history, and any medications you are currently taking and have taken in the past, including any anti-HIV drugs. You will be asked about any other clinical trials you are currently participating in. If you have not had the amount of HIV in your blood measured (a viral load test) within 60 days, you will have about 2 teaspoons of blood drawn from a vein in your arm to find out whether you qualify to take part in this study. Women who are able to become pregnant will either have a urine sample taken or have an additional 1 teaspoonful of blood drawn for a pregnancy test.

Preentry

If you qualify for this study, you will be asked to return to the clinic to have about 1 tablespoon of blood drawn to measure the amount of HIV in your blood and to check your ability to fight infection.

Study Entry and On Study Evaluations

You will be asked to have the following procedures performed on the day you enter the study and at study week 8 and every 8 weeks thereafter until week 96:

Clinical Evaluations and Blood Collection:

You will be asked questions about how you feel and any medications you are taking. At entry, you will have a physical examination performed. You will have about 2 tablespoons of blood drawn to measure the amount of HIV in your blood and to check your ability to fight infection.

Some of the blood drawn will be stored for special tests that will be done later to learn more about the HIV in your blood and how it might change during the study. These tests may include learning how the HIV in your blood is affected by anti-HIV drugs (resistance testing), growing your virus in the laboratory to see how quickly it can grow, and comparing the HIV in your blood to other types of HIV (genetic sequencing). These tests are experimental (not approved by the Food and Drug Administration [FDA]) and have not been proven to have a clinical benefit.

You will be told the results of your HIV viral load tests as part of A5077 as they become available.

For Women Only: If you are able to become pregnant and think you may be pregnant at any time during the study, you will either have a urine sample taken or have an additional 1 teaspoonful of blood drawn for a pregnancy test.

**In addition to the c**linical evaluations and blood **tests listed above, you will be required to give samples of saliva, and you may choose to give samples of either genital secretions or lymphoid tissues, or both, on the day you enter the study and at study weeks 16, 48, and 96. Although it is preferred that you have the same compartments sampled at each visit, you may choose to have genital secretions sampled at one visit and lymphoid tissues at another, or vice versa. These tests are described below.**

Saliva Collection:

The collection of saliva requires that you do not eat or brush your teeth within 30 minutes of collection. You will be asked to spit out about 1 teaspoon of saliva into a sterile tube. This procedure will take about 5-15 minutes.

Genital Secretions Collection:

For Men Only: The collection of genital secretions will require that you do not have any sexual activity for 48 hours prior to the collection. You will need to masturbate without lubricants other than water in a private room at the clinic or at home provided that you can bring your sample to the clinic within 3 hours. You will deposit your semen in a sterile container provided to you.

For Women Only: The collection of genital secretions will require that you do not have any sexual activity and that you do not douche or use any vaginal products in or around your vaginal area for at least 48 hours prior to the collection. The collection of cervical/vaginal fluid will be obtained by inserting a speculum (an instrument that stretches the opening of the vagina) into the vagina and washing the entrance of the cervix (the cervix is the opening of the womb or uterus) with sterile fluid from a syringe. A small plastic tube (catheter) will be inserted into the vagina and fluid will be squeezed into the vagina and towards the cervix. The fluid will then be withdrawn into the same syringe. This procedure will be repeated 2-3 times using the same fluid.

Fluid and cells from your cervix will then be collected. If you are coenrolled in AACTG protocol A5029, you will also have a Pap smear performed.

Lymphoid Tissue Collection:

If you choose to have lymphoid tissues collected, you will either have a tonsil biopsy, lymph node biopsy, lymph node aspiration, or a gut-associated lymphoid tissue (GALT) biopsy. You and your doctor will decide which procedure you will have performed. These procedures are briefly described below. More information will be provided to you before your procedure is performed. You may need to sign a separate informed consent form at the clinic or hospital where the procedure is performed.

Tonsil Biopsy:

A tonsil biopsy is the removal of a small piece of tonsil tissue with a surgical instrument. You will be scheduled to have your biopsy procedure performed in the outpatient clinic by the ear, nose, and throat (ENT) doctor. You will be asked NOT to take any aspirin, nonsteroidal anti-inflammatory drugs (NSAIDs) like Advil and Motrin, or other similar medications 2 weeks before the scheduled tonsil biopsy, because they may prevent your blood from clotting. If you are unsure, ask your study doctor or nurse which drugs to avoid.

During the biopsy procedure, the area around your tonsils in the back of your throat will be made numb by medicine, and the area will be injected with drugs to numb tissue and decrease bleeding. A small instrument will be placed in your mouth to obtain a tissue sample from your tonsils.

The procedure for obtaining the tonsil tissue samples will last about 10 to 15 minutes. You may be asked to wait in the clinic for an extra 30 to 60 minutes in order to check for any excess pain or bleeding from your tonsils.

Lymph Node Biopsy:

A lymph node biopsy is the removal of a lymph node by making a small cut into the skin. The procedure takes 15 to 30 minutes. You will be asked NOT to take any aspirin, NSAIDs like Advil and Motrin, or other similar medications two weeks prior to the scheduled procedure, because they may prevent your blood from clotting. If you are unsure, ask your study doctor or nurse which drugs to avoid. A medication that numbs the lymph node area will be used in order to decrease the risk of discomfort.

Lymph Node Aspiration:

A lymph node aspirate is the removal of a small amount of fluid from a lymph node using a small needle. You will be asked NOT to take any aspirin, NSAIDs like Advil and Motrin, or other similar medications two weeks prior to the scheduled procedure, because they may prevent your blood from clotting. If you are unsure, ask your study doctor or nurse which drugs to avoid. A medication which numbs the node area will be used in order to decrease the risk of discomfort. This procedure will take about 5 to 15 minutes.

GALT Biopsy:

A GALT biopsy is the removal of a small piece of tissue from the bowel with a surgical instrument. Your scheduled biopsy procedure will be performed in the outpatient clinic by a doctor who specializes in the digestive tract (a gastroenterologist). You will be asked NOT to take any aspirin, NSAIDs like Advil and Motrin, or other similar medications two weeks prior to the scheduled biopsy, because they may prevent your blood from clotting. If you are unsure, ask your study doctor or nurse which drugs to avoid.

During the biopsy procedure, a tube (called an endoscope) will be put through your rectum and into your bowel. A mild sedative (a medication that calms or relaxes you, and may make you fall asleep) may be given to you in a vein in your arm before the procedure. A small amount of tissue will be cut from your bowel using a surgical instrument that is passed through the endoscope.

This procedure will last about 30 minutes. You will be asked to wait in the clinic for two hours after the procedure is over to check for any pain or bleeding.

You will not be told the results of the tests in your saliva, genital secretions, or lymphoid tissues until after this study ends.

If the HIV in Your Blood Increases

If the HIV in your blood (your viral load) is greater than or equal to 200 copies at or after 16 weeks on this study, you will be asked to return to the clinic to have about 2 tablespoons of blood drawn to confirm this result. If this result is confirmed, you will be asked to return to the clinic to have all of the tests and procedures that were done at entry performed. You and your doctor will be told your viral load results, and your doctor may recommend changes to your antiretroviral drug regimen. You will remain in this study even if your viral load increases and/or you change your antiretroviral drug regimen.

Early (Premature) Discontinuation

If you decide to leave this study before it ends, you will be asked to return to the clinic to have all of the tests and procedures that were done at entry performed.

OTHER INFORMATION

This study team may need to see the results of certain tests done and information collected during your participation in A5077 from other studies you are enrolled in and from clinic visits, and may access and perform analyses on your blood samples collected for other studies while you are participating in A5077. Information from the other studies and this study will not have your name on it. Your information will be identified by a number to protect your privacy. It is important that you understand that when you sign this consent form, you are also giving the team permission to look at these other records and/or obtain samples of your blood. Information may need to be obtained from your clinic records. Information collected from your clinic records will also be kept confidential and identified by a number to protect your privacy.

Some of your blood and/or tissue obtained as part of this study will be stored, with your identity protected, and used for ACTG-approved AIDS-related research in the future.

RISKS AND/OR DISCOMFORTS

Risks of Drawing Blood

Taking blood may cause some discomfort, bleeding, or bruising and/or swelling where the needle enters the body, and in rare cases, fainting or infection.

Risks of Genital Secretions Collection

The risks of collection of genital secretions (male and female) are pain and discomfort.

Risks of Tonsil Biopsy

The risks of a tonsil biopsy include bleeding, infection, swelling at the site of the biopsy, and pain.

You also may experience an allergic reaction to the medication used to numb your tonsil. This could include itching, hives, swelling, shortness of breath, difficulty breathing, changes in blood pressure, cardiac rhythm changes (changes in your heart beat), loss of consciousness, or death in rare cases.

If you have any pain following the procedure, you may be allowed to take an over-the-counter pain medication. A numbing spray may be allowed to make you more comfortable. The ENT doctor will talk with you about your choices of pain medicine. If excessive pain or bleeding develops, you will be asked to contact the clinic. You may be asked to come to the clinic and be examined by the ENT doctor.

Risks of Lymph Node Biopsy

The risks of a lymph node biopsy include bruising, swelling, bleeding, pain, and/or infection at the site of the biopsy. There is also a small risk that you may have an allergic reaction to the medicine used to numb your skin. This could include itching, hives, swelling, shortness of breath, difficulty breathing, changes in blood pressure, cardiac rhythm changes, loss of consciousness, or death in rare cases.

Risks of Lymph Node Aspiration

The risks of a lymph node aspirate include bruising, swelling, bleeding, pain, and/or infection at the site of the biopsy. There is also a small risk that you may have an allergic reaction to the medicine used to numb your skin. This could include itching, hives, swelling, shortness of breath, difficulty breathing, changes in blood pressure, cardiac rhythm changes, loss of consciousness, or death in rare cases.

Risks of the GALT Biopsy

The risks of a GALT biopsy include discomfort, bleeding, pain, and possible tearing of the bowel. In a small number of cases, bleeding or tearing of the bowel may require surgery to be corrected. If you take a sedative for this procedure, some of the risks of a sedative include drowsiness (sleepiness), dizziness or light-headedness, fatigue (feeling tired), and incoordination (temporary loss of control over your muscles). You should not drive a car while under the effects of a sedative. You will be required to stay in the clinic for at least 2 hours after your procedure is performed. Your doctor may recommend that someone drive you home after the procedure or will request that you stay in the clinic until the effects of the sedative wear off.

PREGNANCY AND BREAST-FEEDING

If you become pregnant during this study, you may continue to take part in this study, but you will only be allowed to have blood and saliva samples collected. You will not be allowed to have genital secretions or lymph tissues collected during your pregnancy. Once your pregnancy is complete, you may have genital secretions and/or lymph tissues collected again.

Women who are breast-feeding may take part in this study.

BENEFITS

There will be no direct benefit to you from being in this study. However, knowledge gained from this study may help others who have HIV/AIDS in the future.

NEW FINDINGS

You will be told of any new information learned during the course of the study that might cause you to change your mind about staying in the study. At the end of the study, you will be told when results of the study will likely be made available and how to learn about them.

REASONS WHY YOU MAY BE WITHDRAWN FROM THE STUDY WITHOUT YOUR CONSENT:

• the investigator decides that continuing in the study would be harmful to you;

• you are not able to follow the requirements of the study;

• the study is cancelled by the National Institute of Allergy and Infectious Diseases;

• other administrative reasons.

COSTS TO YOU

You will not have to pay anything for the clinic visits or tests that are part of this study. Medical costs of other treatment outside of this study will be charged to you or your health insurance company. You [will or will not (sites should choose one)] receive financial payment for taking part in this study. (It is up to each site to choose whether subjects will be paid for their participation in this study. If subjects will be paid, insert the appropriate wording related to when and how the payments will be made).

CONFIDENTIALITY

Your research records will be confidential to the extent permitted by law. You will be identified by a code, and personal information from your records will not be released without your written permission. You will not be personally identified in any publication about this study. However, under guidelines of the Federal Privacy Act, your records may be reviewed by the National Institute of Allergy and Infectious Diseases, Division of AIDS, and by study monitors.

RESEARCH-RELATED INJURY

If you are injured as a result of being in this study, the (name of the clinic) will give you immediate necessary treatment for your injuries. The cost for this treatment will be charged to you or your insurance company. You will then be told where you may receive additional treatment for injuries. There is no program for monetary compensation or other forms of compensation for such injuries.

PROBLEMS OR QUESTIONS

If you ever have questions about this study or in case of research-related injuries, you should contact (name of investigator) at (telephone number), or if you have questions about research subject's rights you can call (name and title of IRB member) at (telephone number).

SIGNATURE PAGE

This is only a suggested signature page. Sites may use their own signature page.

If you have read the informed consent (or if you have had it explained to you) and understand the information, and you voluntarily agree to take part in this study, please sign your name below.

Volunteer's Name Volunteer's Signature Date

(typed or printed)

OR OR

Volunteer's Legal Guardian Legal Guardian's Signature Date

or Representative

Witness' Name Witness' Signature Date

(typed or printed)

NOTE: This consent form with the original signatures MUST be retained on file by the principal investigator. A copy must be given to the volunteer. A copy should be placed in the volunteer’s medical record, if applicable.

The Division of AIDS strongly encourages a witness for the volunteer's signature.
